# An Acute Stress Model in New Zealand White Rabbits Exhibits Altered Immune Response to Infection with West Nile Virus

**DOI:** 10.3390/pathogens8040195

**Published:** 2019-10-18

**Authors:** Willy W. Suen, Mitchell Imoda, Albert W. Thomas, Nur N.B.M. Nasir, Nawaporn Tearnsing, Wenqi Wang, Helle Bielefeldt-Ohmann

**Affiliations:** 1 School of Chemistry & Molecular Biosciences, The University of Queensland, St Lucia, Qld 4072, Australia; willy.suen@csiro.au; 2 School of Veterinary Science, The University of Queensland, Gatton, Qld 4343, Australia; Mitchell.imoda@gmail.com (M.I.); albert.w.thomas@gmail.com (A.W.T.); n.tearnsing@uq.net.au (N.T.); wgwenqi@hotmail.com (W.W.); 3 School of Biomedical Sciences, The University of Queensland, St Lucia, Qld 4072, Australia; nurnamirahbinte.mohdnasir@uq.net.au; 4 Australian Infectious Diseases Research Centre, The University of Queensland, St. Lucia, Qld 4072, Australia

**Keywords:** West Nile virus, immunosuppression, dexamethasone, rabbit model, transcriptome, antiviral response

## Abstract

The immune competence of an individual is a major determinant of morbidity in West Nile virus (WNV)-infection. Previously, we showed that immunocompetent New Zealand White rabbits (NZWRs; *Oryctolagus cuniculus*) are phenotypically resistant to WNV-induced disease, thus presenting a suitable model for study of virus-control mechanisms. The current study used corticosteroid-treated NZWRs to model acute “stress”-related immunosuppression. Maximal effects on immune parameters were observed on day 3 post dexamethasone-treatment (pdt). However, contrary to our hypothesis, intradermal WNV challenge at this time pdt produced significantly lower viremia 1 day post-infection (dpi) compared to untreated controls, suggestive of changes to antiviral control mechanisms. To examine this further, RNAseq was performed on RNA extracted from draining lymph node—the first site of virus replication and immune detection. Unaffected by dexamethasone-treatment, an early antiviral response, primarily via interferon (IFN)-I, and induction of a range of known and novel IFN-stimulated genes, was observed. However, treatment was associated with expression of a different repertoire of IFN-α-21-like and IFN-ω-1-like subtypes on 1 dpi, which may have driven the different chemokine response on 3 dpi. Ongoing expression of Toll-like receptor-3 and transmembrane protein-173/STING likely contributed to signaling of the treatment-independent IFN-I response. Two novel genes (putative HERC6 and IFIT1B genes), and the SLC16A5 gene were also highlighted as important component of the transcriptomic response. Therefore, the current study shows that rabbits are capable of restricting WNV replication and dissemination by known and novel robust antiviral mechanisms despite environmental challenges such as stress.

## 1. Introduction

West Nile virus (WNV) is a mosquito-borne flavivirus that has the potential to cause fatal encephalitis in humans, horses, birds and several other animals. WNV is a pathogen of global significance with disease outbreaks occurring in Europe, Africa, the Middle East and the Americas. Since its first incursion into North-America in 1999, more than 50,000 human cases have occurred in the USA alone with more than 2300 fatalities [1]. Until recently, highly virulent strains of WNV have been exotic to Australia, although a strain of low virulence (Kunjin-KUNV) has circulated here for more than 50 years, causing only rare cases of mild clinical disease in humans and horses [2,3]. This scenario changed dramatically in 2011 when an unprecedented outbreak of equine WNV encephalitis occurred in south eastern Australia. More than 1000 horses were affected, with a mortality rate of 10–15% [4]. Molecular and antigenic analysis of the virus responsible for the outbreak, named WNV_NSW2011_, revealed it was most closely related to KUNV, indicating it was of local origin [4]. Interestingly, no severe human clinical cases were associated with this outbreak, despite low levels of pre-existing immunity in the human population [2].

It is estimated that ~80% of all WNV infections in humans are subclinical [5,6]. Asymptomatic infection may be due to pre-existing immunity (prior exposure or cross-reactive immunity) or inherent ‘resistance’. Severe illness in WNV infection (hospitalization, neuroinvasive disease and death) is often associated with co-morbidities, notably, immunosuppression, diabetes and hypertension [6,7,8]. Thus far, the WNV research focus has largely been on the mechanisms of neuroinvasive disease using mouse models [9,10], while the mechanisms underlying “resistance” and the effect of co-morbidities remain poorly understood. Yet, it is exactly those mechanisms, most likely relating to innate immune reactions and immune dysregulation, respectively, that should be targeted in the development of new vaccines and adjuvants [11,12,13,14]. The much-exaggerated level of morbidity and mortality in mice following WNV-infection suggests a potentially different pathogenesis of disease in this species compared to humans and horses [10,15,16,17], and call into question the utility of the mouse models for study of human [18,19] and equine [17] WNV infections. We have developed a rabbit model of non-lethal WNV infection, which mimics the course of the majority of human and equine WNV-infections, including low, transient viremia, and no or negligible morbidity despite histopathological evidence of infection-induced neuro-inflammation and virus replication in peripheral tissues [17,20,21].

It has long been known that “stress”, whatever its proximal cause, can have severe adverse effects on innate resistance to microbial infections. Nutritional deficiencies, long-distance transport, co-infections with infectious agents of any category and treatment with anti-inflammatory drugs, or any combination of these settings, can generate the conditions in a host which adversely affect its ability to resist and eliminate infectious agents [22]. The use of corticosteroids is widespread in treatment regimens to alleviate inflammatory and allergic conditions, as well as in transplant patients to abrogate transplant rejection and in chemotherapeutic regimens. However, its negative effects on immune functions contribute to increased risk of infections [23,24,25].

In this study, we examined the effect of acute “stress”, in the form of a single systemic injection of a synthetic corticosteroid drug, dexamethasone, on viral replication and the antiviral innate immune response following intradermal WNV infection in young rabbits. Contrary to our hypothesis, intradermal WNV infection on day 3 post-dexamethasone treatment (pdt) produced significantly lower viremia in treated rabbits relative to that in the untreated controls. This may—at least in part—be explained by differences in the post infection transcriptome of antiviral effector molecules in treated versus non-treated animals.

## 2. Results

### 2.1. Characterization of the Acute Stress Model Using Bolus Administration of Dexamethasone

Based on previous studies using dexamethasone administration to model stress [26], we adopted a similar protocol for the current study. As a pilot study to validate the changes in immune responses associated with dexamethasone treatment and to investigate the optimal time post-dexamethasone treatment for WNV inoculation in the subsequent study, four NZWRs of approximately 4–5 weeks of age were injected with 6.5 mg/kg of dexamethasone intramuscularly and bled once prior to treatment and daily thereafter for 7 days. Two NZWRs of the same age were used as controls. On day 3 and 7 pdt, two treated and one untreated control were culled, with tissue harvested.

As expected, [27], a single dose of dexamethasone treatment resulted in a shift in the peripheral blood leukogram, with decreased percentage of lymphocytes and increased heterophils (the rabbit equivalent of neutrophils) (Supplementary Figure S1). The peak of this change occurred on day 3 pdt (Supplementary Figure S1B,C).

Total RNA was extracted from whole blood and the relative abundance of transcripts of selected cytokine transcripts was assessed by quantitative reverse-transcription polymerase chain reaction (qRT-PCR). While the levels of these cytokine transcripts were mostly comparable for all time-points assessed (day 0, 1, 3 and 7 pdt), a significant difference was observed for interferon-alpha (IFN-α) transcript on day 3 pdt (p < 0.05), where the treated group had significantly lower levels of this transcript relative to that in the untreated control (Supplementary Figure S2). Expression was restored to baseline level by day 7 pdt.

At day 3 pdt, the thymic cortex had decreased markedly in the dexamethasone-treated animals compared to the control animal (Supplementary Figure S3). Immunolabeling for Ki67 and activated caspase 3 revealed that the decreased cell numbers was likely due to decreased cell proliferation rather than cell death by apoptosis, and this interpretation was corroborated by the absence of increased numbers of macrophages with apoptotic bodies or other histological evidence of cell demise (data not shown). Similarly, there was mild reduction in cell densities in some lymph nodes of the dexamethasone treated animals at day 3 pdt, but by day 7 pdt the effect had largely been reversed both in the thymus and in the lymph nodes (Supplementary Figure S3 and data not shown).

### 2.2. Use of Acute Stress Model for WNV Infection

Sixteen rabbits, 4–5 weeks old, were randomly allocated to the mock-treatment (phosphate-buffered saline (PBS); n = 7) and dexamethasone-treatment (n = 9) groups, respectively. Three days after the PBS or dexamethasone-administration, they were challenged intradermally in the left hind-footpad with 50 µL medium containing 10^5^ tissue culture infective dose 50% end-point (TCID_50_) units of WNV_NSW2011_ as per the previously described WNV challenge model [20]. The animals were monitored and bled daily, and scheduled culls and necropsies performed on days 1, 3 and 7 post-infection (p.i.; further details in Section 4).

All animals grew at a normal rate during the 10 days experimental period. Only brief, intermittent fever-spikes were noted on day 1 p.i., and none of the animals showed neurological signs or any other serious adverse effects of either the dexamethasone treatment or the virus infection. Swelling of the left popliteal lymph nodes were noted in most of the infected animals regardless of pre-treatment and this enlargement was also evident at necropsy, both of the left (draining) and, in some animals, the right popliteal lymph node. All other organs and tissues were grossly unremarkable, including the viral injection site in the left footpad.

#### 2.2.1. Hematological Parameters in Untreated and Dexamethasone-Treated WNV-Infected Rabbits.

**Figure 1:**
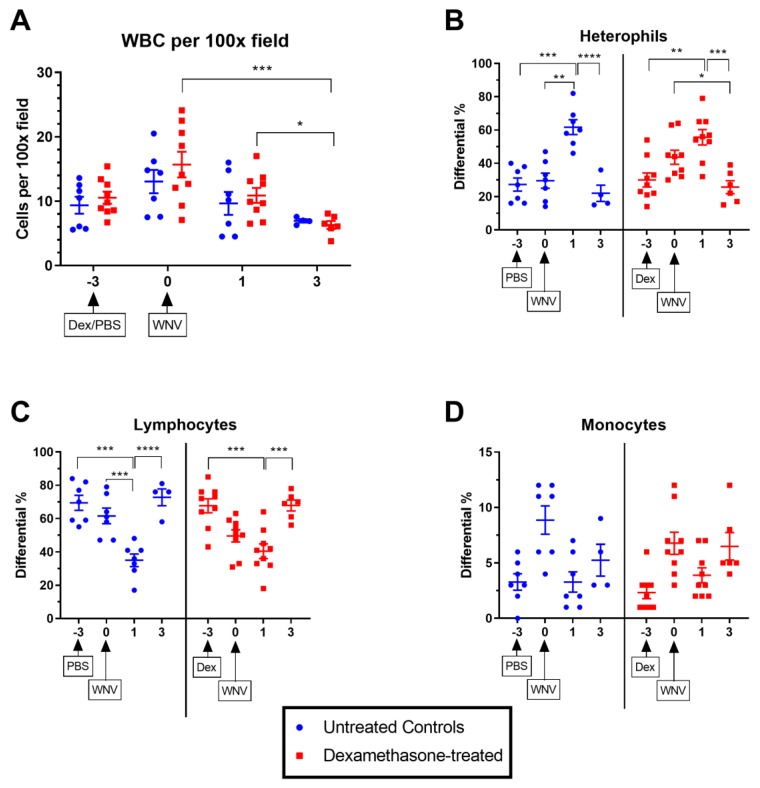
Hematological profile in mock- and dexamethasone-treated, WNV-infected rabbits on days -3 to 3 post-infection (p.i.). (**A**) Total number of white blood cells (WBC) in a 100x field, (**B**) Percentage heterophils, (**C**) Percentage lymphocytes, (**D**) Percentage monocytes. Statistical significance was determined by two-way analysis of variance (ANOVA). *, *p* = 0.01–0.05; **, *p* = 0.001–0.01; ***, *p* = 0.0001–0.001; ****, *p* < 0.0001. Each data point represents one rabbit in each group. The error bars represent the standard error of the mean and the middle bar represents the mean.

To evaluate the hematological changes associated with dexamethasone administration, leukocyte counts were again performed on blood smears prepared from blood samples collected prior to dexamethasone/PBS treatment, immediately prior to WNV infection (day 3 pdt), and day 1 and 3 post-WNV challenge. On day 3 pdt (day 0 p.i.), a stress leukogram was observed in the dexamethasone treated group, where there was an increase in the heterophilic and a decrease in the lymphocytic population as compared to those prior to treatment (Figure 1). Moreover, the stress leukogram was further magnified on day 1 post-WNV challenge, regardless of dexamethasone treatment. The leukocyte proportions returned to baseline on day 3 p.i., consistent with the asymptomatic clinical course (Figure 1).

#### 2.2.2. Virus Kinetics in Untreated & Dexamethasone-Treated WNV-Infected Rabbits

**Figure 2:**
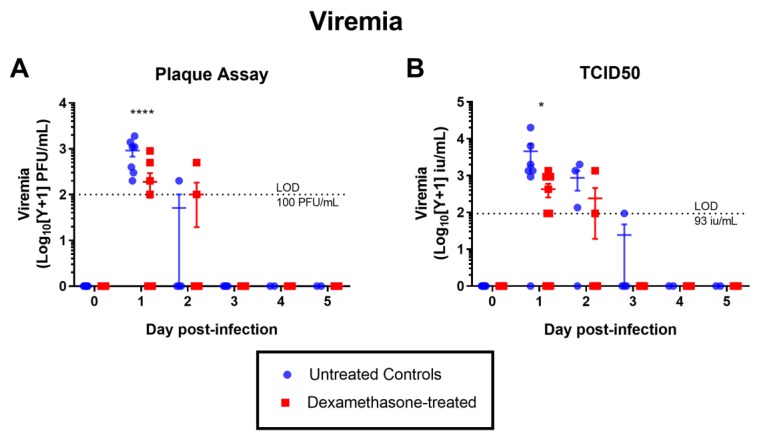
Level of viremia in untreated and dexamethasone pre-treated rabbits challenged with 10^5^ TCID_50_ of WNV by injection in the left footpad. Virus loads in serum were measured by (**A**) plaque assay and (**B**) TCID_50_. LOD = limit of detection. Statistical significance was determined by two-way ANOVA. *, *p* = 0.01–0.05; **, *p* = 0.001–0.01; ***, *p* = 0.0001–0.001; ****, *p* < 0.0001. Each point represents the viremic titer of each of the rabbits in each group. The error bars represent the standard error of the mean and the middle bar represents the mean. Note that titers below the LOD are represented as zero.

**Figure 3:**
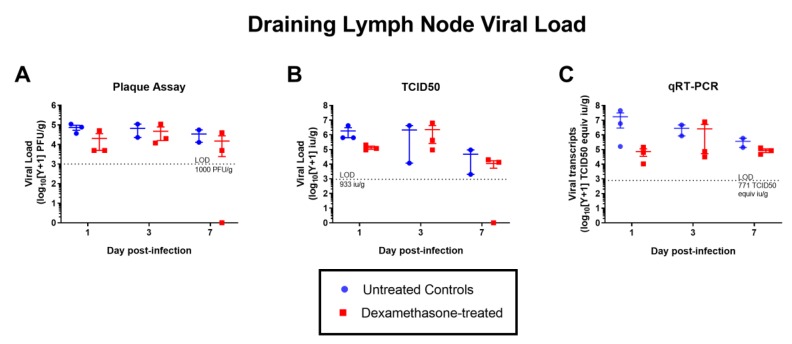
Viral load in the left popliteal lymph node (draining the footpad injection site) in untreated and dexamethasone pre-treated rabbits, measured by three different approaches: (**A**) plaque assay, (**B**) TCID_50_ assay, (**C**). qRT-PCR. LOD = limit of detection. No statistical significance was detected when groups were compared by two-way ANOVA. Each point represents the viral load of each of the rabbits in each group. The error bars represent the standard error of the mean and the middle bar represents the mean. Note that titers below the LOD are represented as zero.

**Figure 4:**
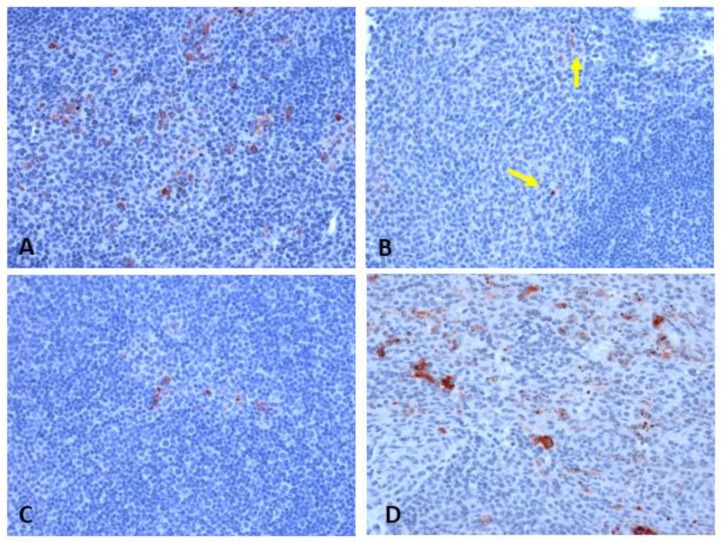
Immunohistochemical detection of WNV antigen (NS1; red cells) in left popliteal lymph node of (**A**) non-treated rabbit day 1 p.i. (score of +++), (**B**) dexamethasone-treated rabbit day 1 p.i. (yellow arrows point to rare, scattered NS1-positive cells; score of +), (**C**) non-treated rabbit day 3 p.i. (score of +), (**D**) dexamethasone-treated rabbit day 3 p.i. (score of ++). Original magnification 200×.

Contrary to expectations, the viremia in the dexamethasone-treated group was significantly lower on day 1 p.i. compared to the mock-treated group. This was consistent across the two methods of virus titration, plaque assay and tissue culture infective doses at 50% cut-off (TCID_50_) (Figure 2). There was no statistically significant difference in viral load in the left popliteal lymph node draining the site of inoculation (hereafter referred to as the draining lymph node) between the two groups (Figure 3). This was also consistent across the three methods of virus titration performed (plaque assay, TCID_50_, and qRT-PCR). By immunohistochemistry (IHC), we assessed the presence of WNV-NS1 antigen in the draining lymph node and other lymphoid and non-lymphoid organs (Table 1) as an indication of the extent of virus dissemination to distant sites. While in most rabbits viral antigen was largely restricted to the draining lymph node, one dexamethasone-treated rabbit (2105) had detectable antigen in several of the distant organs (Table 1). Another rabbit of the same group also had detectable antigen in the spleen, in addition to the draining lymph node (Table 1). The majority of NS1-positive cells were large pleomorphic leukocytes, with morphology consistent with dendritic cell and macrophage-like cells (Figure 4). By day 7 p.i., no infected cells were detected by IHC in the draining lymph node, despite a low viral titer on virus isolation and qRT-PCR (Figure 3). This is likely explained by the difference in the sensitivity of the assays, where the amount of tissue used in the homogenates for virus isolation/qRT-PCR provided higher sensitivity than that represented by the 4 µm thick tissue sections used for IHC.

#### 2.2.3. Histopathology and Immunophenotyping of Lymphoid Organs

Samples from lymphoid organs (thymus, spleen and lymph nodes), injection site in the footpad, brain (multiple sites) and liver were examined by microscopy. The most notable changes were present in the left popliteal lymph node—the lymph node draining the injection site—where very prominent hypertrophy of the post-capillary venules was evident from day 1 p.i. in the mock-treated animals and from day 3 p.i. in the dexamethasone-treated animals. Development of secondary follicles was also evident by day 7 p.i. for both treated and untreated animals. In contrast, a decrease in the thymic cortex, similar to that seen in the preliminary study (Supplementary Figure S3) was evident at day 1 and 3 p.i. in the dexamethasone treated animals, but had resolved by day 7 p.i. This change was not evident in any of the mock-treated animals at any time point p.i. Similarly, a slight decrease in the white pulp of the spleen was evident in the day 1 and 3 p.i. dexamethasone-treated animals, but this was followed by reconstitution and hypertrophy by day 7 p.i. Mild inflammation in the injection site was only detected in one mock-treated animal on day 7 p.i., but it cannot be excluded that similar reactions were present in other animals, but missed as only a few 4 µm sections were examined for each animal.

Immunolabeling for T lymphocytes (CD3), B lymphocytes (CD79a) and myeloid-lineage cells (macrophages and heterophils; Mac387) did not reveal any major or consistent differences in relative numbers and distribution between the mock- and dexamethasone-treated, WNV-infected animals for any of those cell populations at any time point (data not shown).

#### 2.2.4. Antibody Responses

Sera from animals culled on day 1 and 7 p.i. were tested by blocking-ELISA and virus neutralization test (VNT) for antibodies specific for WNV. All day 1 p.i. sera were negative in both assays, while all five (three dexamethasone-treated and two PBS-treated) of the day 7 p.i. sera were positive for flavivirus E-protein specific antibodies in the blocking-ELISA. Of these, three had WNV-neutralizing activity: one dexamethasone-treated animal had a titer of 1:40 and the two PBS-pretreated animals had titers of 1:20 and 1:40, respectively. All day 7 p.i. sera were negative for WNV-NS1 specific antibodies in the blocking-ELISA, suggesting that virus replication was quickly contained before release of substantial amounts of NS1-protein.

#### 2.2.5. Differential Gene Expression Analyses of RNA Extracted from Fraining Lymph Nodes

In our previous studies, we demonstrated the draining popliteal lymph node was the first tissue site with detectable tissue viral load [20,21], and thus, a suitable site for study of early antiviral responses. Therefore, RNA was extracted from the draining lymph node and RNAseq was performed in order to examine potential differences in the early immune response between dexamethasone and mock-treated rabbits. Table 2 summarizes the denotation assigned to each of the groups for downstream differential gene expression and gene ontology (GO) analyses. Two mock-treated, non-infected animals from the pilot study were also included (denoted C0 in Table 2).

Of the 16 WNV-infected rabbits (nine dexamethasone-treated and seven mock), one RNA sample (from animal 2103) was excluded for RNAseq, due to an unworkably low RNA integrity number (RIN score = 3.3). The RIN score for the remaining samples ranged from 4.9–6.8, and an adjustment to the fragmentation time was used during the sequencing library preparation in order to account for the generally low RIN score (see Methods section for more details).

RNAseq of the 17 RNA samples yielded an average of 29067807 reads (range: 22019057–37133333), with all fastq files passing sequence quality check using the Galaxy application, FastQC (Galaxy version 0.72). Pairwise comparisons of transcriptome were performed between dexamethasone-treated WNV-infected groups (T) and untreated-uninfected controls (C); between untreated WNV-infected groups (N) and C; and between T and N. We employed a highly stringent protocol for differential gene expression analysis that used three R-based packages (edgeR, DEseq2, and Voom-limma) under the Galaxy tool “Differential _Count models using BioConductor packages” (Galaxy Version 0.28). Only differentially expressed genes (DEGs) that intersected across all three packages were used for downstream analyses.

**Figure 5:**
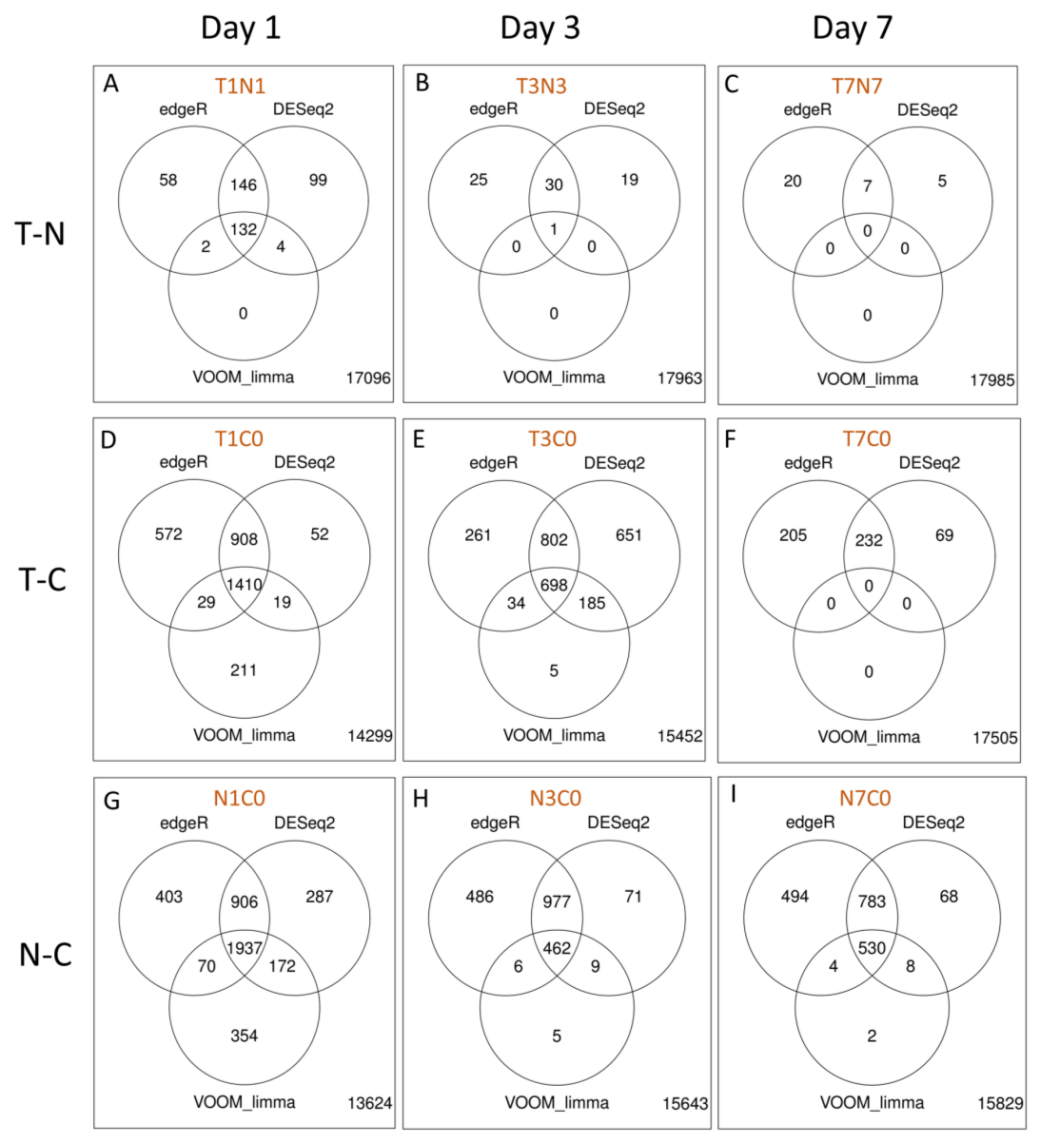
Pairwise comparisons for differential gene expression analyses using edgeR, DESeq2 and Voom-limma. (**A**) T1N1, (**B**) T3N3, (**C**) T7N7, (**D**) T1C0, (**E**) T3C0, (**F**) T7C0, (**G**) N1C0, (**H**) N3C0, and (**I**) N7C0. The days in the column headings refer to the day p.i. The numbers within circles represent the number of DEGs that were common (intersects) between each of the R package analyses or unique to each of the analyses (non-overlapping regions). The number external to the three circles represents the number of genes that were not differentially expressed. Each of the comparisons is denoted by a code (in brown) where the alphabets represent the treatment/infected status, and the number represents the day post-infection. T, dexamethasone treated WNV-infect groups; N, mock-treated WNV-infected groups; C, untreated uninfected group.

The number of intersecting DEGs was highest on day 1 p.i., irrespective of the comparisons (Figure 5A,D,G), suggesting early transcriptional activity is associated with WNV_NSW2011_ infection. The direct comparisons between treated and untreated WNV infected groups (T-N) showed only low numbers of DEGs, the highest number being observed on day 1 p.i. (132 DEGs, Figure 5G). The transcriptome of the draining lymph nodes of rabbits treated with dexamethasone and infected with WNV_NSW2011_ was generally comparable with that of mock-treated rabbits, at least for day 3 and 7 p.i. In order to investigate the transcriptional dynamics of genes associated with WNV_NSW2011_ restriction, pairwise differential gene expression analyses were performed for each of the groups, comparing against the mock-treated uninfected controls. Consistent with the T-N comparisons, the number of DEGs between T-C and N-C comparisons was generally comparable for day 1 and 3 p.i. (1410 versus 1937 on day 1 p.i.; 698 versus 462 on day 3 p.i.; Figure 5D,E,G,H). A notable difference was observed on day 7 p.i., however, where only the N-C comparison yielded DEGs (530 DEGs; Figure 5I). No DEG was identified in the T7C0 comparison (Figure 5F).

#### 2.2.6. Gene Ontology Analyses

The intersecting DEGs from each analysis were assessed for enrichment of functional annotation, using the gene ontology (GO) tool, DAVID 6.8. An adjusted *p*-value threshold of 0.05 using the Benjamini correction was set as the cut-off for GO term enrichment. Enriched GO terms from each of the pairwise comparisons were then analyzed for commonalities and differences using Venn diagrams (Supplementary Figure S4). Across all the comparisons against the control group that yielded DEGs (i.e. T1C0, T3C0, N1C0, N3C0, and N7C0, Figure 5), two GO terms “defense response to virus” (GO: 0051607) and “extracellular exosome” (GO: 0070062) were consistently enriched, highlighting the importance of these two annotations in the virus challenged groups regardless of dexamethasone treatment and time post-infection.

On day 1 p.i. and across the treated and mock comparisons against controls, other commonly enriched GO terms relevant to the context of virus control included “negative regulation of viral genome replication” (GO:0045071), “positive regulation of JNK cascade” (GO:0046330) and terms associated with various cellular compartments (Table 3, Supplementary Figure S4). Unique to the DEGs from the T1C0 comparison, there were a further 20 enriched GO terms, many of which were associated with immune response, in particular ones relating to response to lipopolysaccharide, ATP binding, innate immunity, tumor necrosis factor production, macrophage/monocyte chemotaxis, nuclear factor-κB (NF-κB) activity, interleukin-1β (IL-1β) secretion, integrin signaling and IL-8 production (Table 3).

On day 3 p.i., intersecting GO terms across treated and mock-treated WNV-infected rabbits that were within the theme of antiviral activity and immune response included “double-stranded RNA binding” (GO:0003725), “immune response” (GO:0006955), and “ATP binding” (GO:0005524), as well as “defense response against virus” and “extracellular exosome” as mentioned above. On day 7 p.i., only the comparison N7C0 yielded DEGs. GO terms enriched within the N7C0 DEG list that were relevant for further analyses included “defense response to virus” and “extracellular exosome”, which were enriched in all of the other comparisons as mentioned above.

It should be noted that for the following downstream analyses, we excluded several enriched GO terms relating to translation and various intracellular compartments, as their links to antiviral mechanisms seems to be indirect, and it is not expected that they be relevant in resistance to infection.

#### 2.2.7. Antiviral DEGs in Rabbits Infected with WNV_NSW2011_

**Figure 6:**
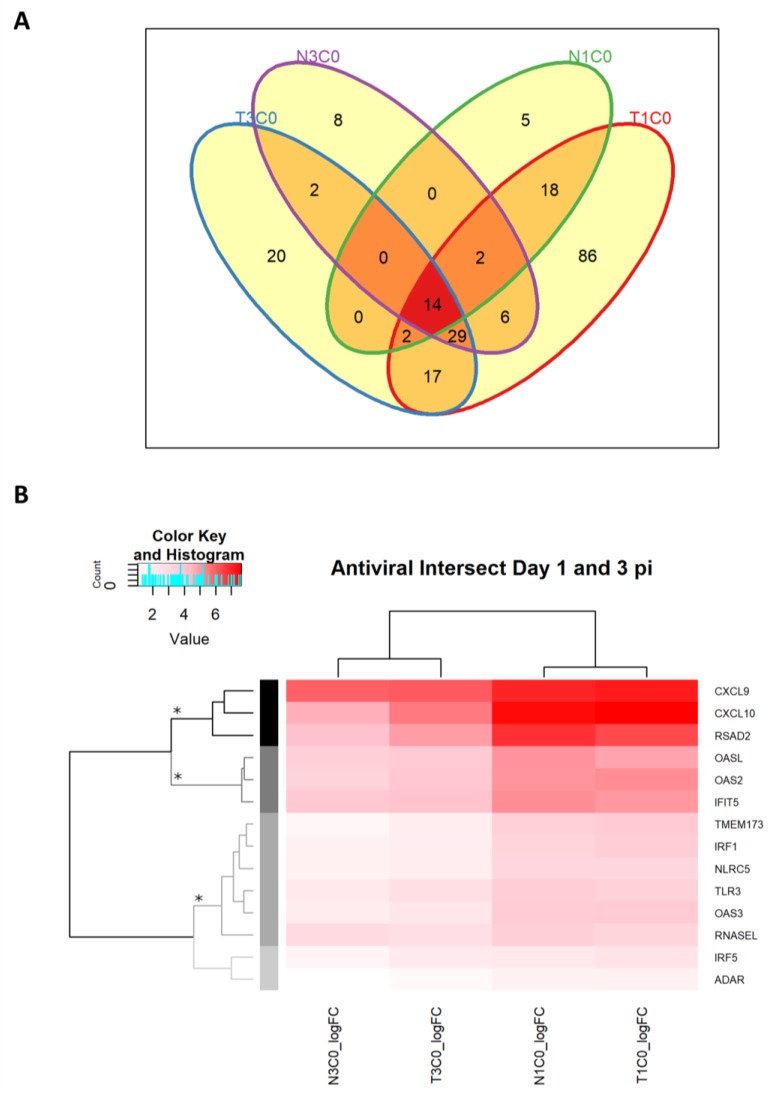

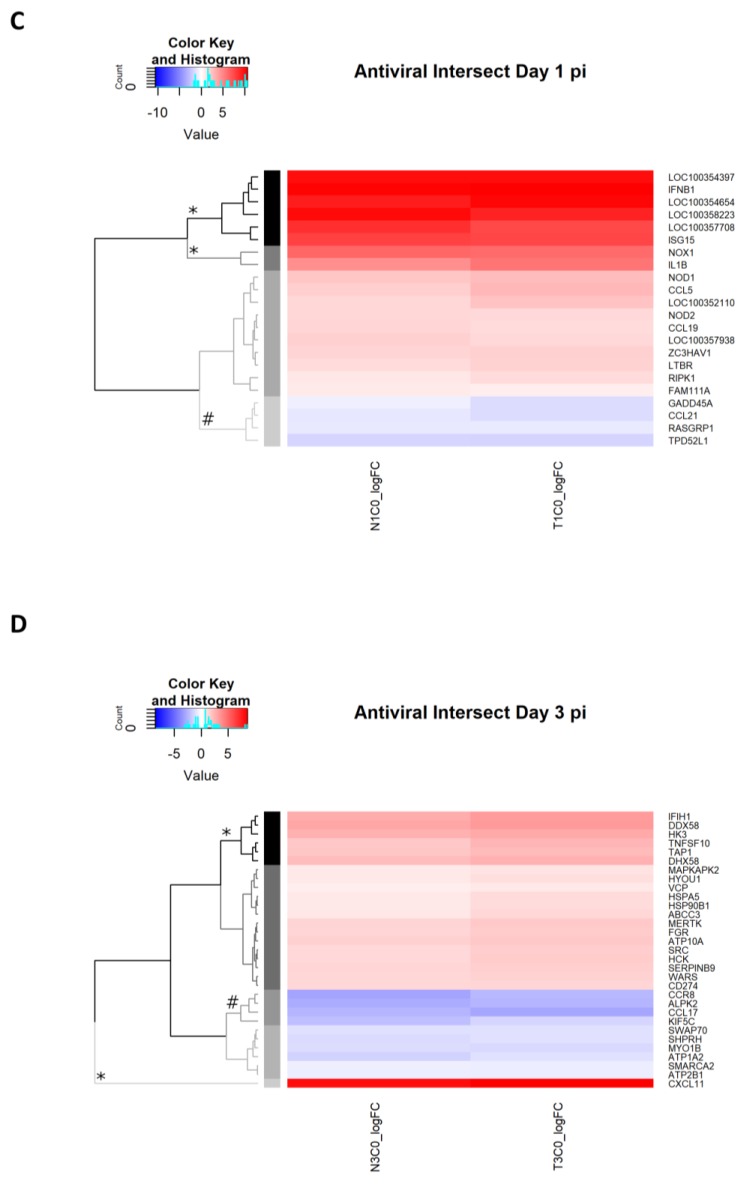

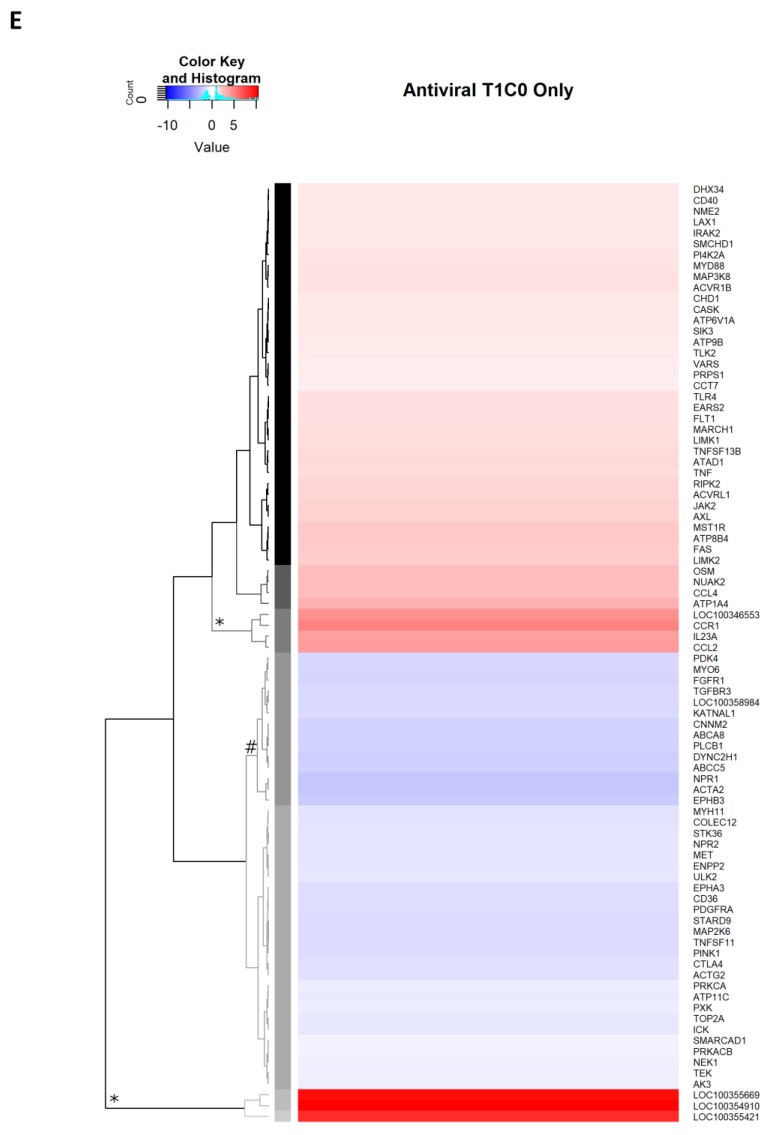

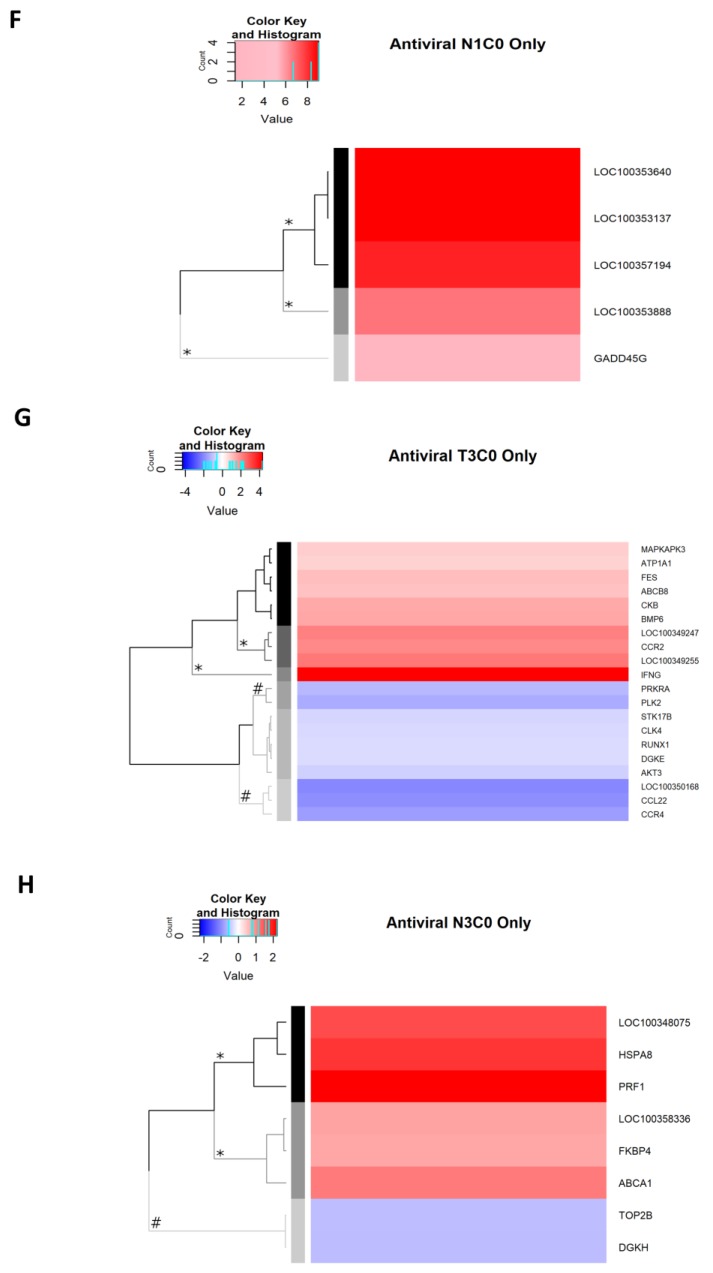
Differentially expressed genes (DEGs) associated with GO terms relevant to antiviral immune responses on day 1 and 3 p.i. (**A**) Venn diagram showing the overlapping DEGs between the T1C0, T3C0, N1C0 and N3C0 comparisons, as well as the unique DEGs. Common and unique DEGs from this Venn diagram are then analyzed for top up- and downregulated DEGs by hierarchical clustering (**B–H**). The grey scale dendrogram branches and the corresponding sidebar in each heatmap represents the different clusters. Clusters with symbols * and # indicate top up- and downregulated clusters, respectively, summarized in Table 4, Table 5 and Table 6. The blue-red scalebar in the top left corner of each heatmap corresponds to the color scale of the heatmap, indicating the logFC for each gene. The light blue histogram within these scalebars indicate the frequency of genes at each level of logFC.

In order to identify and compare which DEG contributed most to enriched GO terms associated with antiviral immune responses on day 1 and 3 p.i., DEG lists from each of the GO terms outlined in Table 3 (excluding ones within the “extracellular exosome” term which were analyzed separately) were compared for similarities and differences (Figure 6A). A total of 209 DEGs were associated with the antiviral GO terms; 14 of which were common to the day 1 and 3 p.i. analyses regardless of dexamethasone-treatment (Figure 6A,B); 22 were uniquely common to only the comparisons on day 1 p.i. (Figure 6A,C); and 31 were uniquely common to only the comparisons on day 3 p.i. (Figure 6A,D). Notably, there were 86 antiviral DEGs that were only expressed in the T1C0 comparison, indicating a higher number of unique DEGs in dexamethasone-treated rabbits on day 1 p.i., as compared to the number of unique DEGs in the remaining groups (Figure 6E). DEGs unique to the comparisons, N1C0, T3C0 and N3C0, are illustrated in Figure 6F,G,H, respectively.

To assess the magnitude of up/downregulation of the antiviral DEGs, heatmaps of the DEG fold change (log_2_FC) were generated (Figure 6B–H, and Supplementary Figure S5) based on subsets of the various intersects of interest in the Venn diagram (Figure 6A). Hierarchical clustering of the logFC was performed for each heatmap in order to identify top upregulated and downregulated clusters of DEGs, which are summarized in Table 4, Table 5 and Table 6, and Supplementary Table S1.

#### 2.2.8. Antiviral Genes Across Day 1 and 3 p.i. Unaffected by Dexamethasone Treatment

Amongst the 14 antiviral DEGs that were similarly differentially expressed across all comparisons on day 1 and 3 p.i., the chemokine genes CXCL9 and CXCL10, and the IFN-stimulated gene (ISG), RSAD2 (also known as viperin), were clustered as DEGs with the highest logFC on day 1 and 3 p.i. (Figure 6B). The second cluster to follow this included the ISGs: OASL, OAS2 and IFIT5 (Figure 6B). Of lower magnitude but nevertheless upregulated in their expression across the day 1 and 3 p.i. groups were the pattern recognition receptors, Toll-like receptor (TLR)-3 and transmembrane protein (TMEM)-173 (also known as stimulator of IFN genes [STING]); the IFN signaling molecule, IFN regulatory factor (IRF)-1 and NOD-like receptor (NLR) family CARD domain containing 5 (NLRC5); and further ISGs such as OAS3, and RNaseL.

The persistent expression of these genes across the groups during these early time-points highlights their significance in a conserved, early antiviral response to WNV_NSW2011_ infection, irrespective of dexamethasone treatment.

#### 2.2.9. Antiviral Genes Uniquely Expressed on Day 1 p.i.

Many of the uniquely upregulated DEGs of the highest magnitude (top clusters) on day 1 p.i. were type I IFN genes. Conserved across the treated and mock-treated analyses on day 1 p.i., top upregulated genes included IFN-β1, IFN-α-21-like and IFN-ω-1-like subtypes at specific loci (IFN-ω-1-like: LOC100354397, LOC100358223; and IFN-α-1-like: LOC100354654, LOC100357708). Notably, however, dexamethasone treatment altered the profile of other IFN-α-21-like and IFN-ω-1-like subtype expression (Table 4). Specific loci for IFN-α-21-like (LOC100355669), and IFN-ω-1-like (LOC100354910 and LOC100355421) were upregulated in dexamethasone treated rabbits, whereas IFN-α-21-like locus, LOC100357194, and IFN-ω-1-like loci, LOC100353640, LOC100353137 and LOC100353888, were upregulated specifically in mock-treated rabbits (Table 4). Other genes that are well-known to be upregulated with early viral infection such as ISG15 and interleukin (IL)-1β were also commonly upregulated on day 1 p.i. along with NADPH oxidase 1 (NOX1).

Unique to dexamethasone treated rabbits on day 1 p.i., there were upregulated expression of the T-helper type I response cytokine IL-23A; chemokine receptor CCR1 and ligand CCL2; and a transmembrane ATP-binding protein, multidrug resistance-associated protein 1 (LOC100346553; also known as ABCC1). Notably, also unique to treated rabbits on day 1 p.i., there were several downregulated genes, in particular ones with nucleotide/ATP binding function (ABCA8, ABCC5, LOC100358984, NPR1, CNNM2, MYO6, KATNAL1, FGFR1, and EPHB3); some of which are transmembrane receptors (e.g. TGFBR3, FGFR1 and EPHB3) (Table 5 and Supplementary Figures S3 and S4). Unique to mock-treated rabbits on day 1 p.i., the expression of growth arrest and DNA damage inducible gamma (GADD45G) gene was upregulated (Table 5).

#### 2.2.10. Antiviral Genes Uniquely Expressed on Day 3 p.i.

On day 3 p.i., common across the treated/mock-treated analyses, the interferon-inducible T cell chemokine, CXCL11, was the top upregulated gene (Figure 6D), followed by few genes that encode proteins with double-stranded RNA binding function, such as DExH-box helicase 58 (DHX58), DExD/H-box helicase 58 (DDX58; also known as retinoic acid-inducible gene I [RIG-I]), and IFN induced with helicase C domain 1 (IFIH1; also known as melanoma differentiation-associated protein 5 [MDA5]). The latter two are well-known pattern recognition receptors (PRRs) that detect cytosolic dsRNA. The tumor necrosis factor superfamily member 10 (TNFSF10, also known as TRAIL) and the ISG, transporter 1 ATP binding cassette subfamily B member (TAP1) were also in the second top cluster of DEGs (Table 6). Concurrently, all day 3 p.i. infected groups had downregulated expression of the chemokine CCL17 and its receptor CCR8, and ATP-binding proteins Alpha Kinase 2 (ALPK2) and Kinesin Family Member 5C (KIF5C; Table 6).

Unique to the dexamethasone treated group on day 3 p.i., expression of IFN-γ was upregulated to the highest extent (Figure 6G and Table 6). Chemokine genes, lymphotactin and CCR2, were also upregulated along with HLA class I histocompatibility antigen B-7 alpha chain. Most prominent downregulated expression was observed for HLA class II histocompatibility antigen DRB1-4 beta chain, and the chemokine ligand CCL22 and its receptor CCR4 (Table 6).

On the other hand, the top upregulated DEGs in the mock-treated rabbits included the GTP-binding tubulin alpha-1B chain-like molecule, the pore-forming cytotoxic perforin 1 (PRF1), and heat shock protein family A (Hsp70) member 8 (HSPA8; Table 6). To a lesser extent, the second cluster of top upregulated genes included the ATP-binding, actin-related protein 3-like protein (LOC100358336), the immunomodulatory FKBP Prolyl Isomerase 4 (FKBP4), and the ATP-binding cassette transporter ABCA1 (Table 6). Specific to the mock-treated group, the expression of DNA topoisomerase II beta (TOP2B) and diacylglycerol kinase eta (DGKH) genes were downregulated to the highest extent (Table 6).

#### 2.2.11. Antiviral Genes Expressed on Day 7 p.i.

On day 7 p.i., the draining lymph node transcriptome of the dexamethasone-treated group was comparable to that of the baseline control, yielding no DEG (Figure 5F). On the other hand, 530 DEGs were identified in the untreated group to control comparison (N7C0; Figure 5I). This suggests that the treated group returned to baseline gene expression faster than mock-treated rabbits, despite similar levels of viral load in the lymph node by this time (Figure 3). Similar to the comparisons in the earlier time-points, the two GO terms “defense response to virus” and “extracellular exosome” were enriched (Supplementary Figure S4C). Within the top upregulated cluster of DEGs in the former term, DEGs that have been featured in earlier comparisons included ISG15 and CXCL9 (Supplementary Figure S5). The chemokine CCL5 and cytidine/uridine monophosphate kinase 2 (CMPK2) were also among the top clustered DEGs (Supplementary Figure S5). Notably, ATP-binding cassette subfamily A member 3 (LOC100353012) was the top downregulated DEG (Supplementary Figure S5).

#### 2.2.12. Extracellular Exosome

**Figure 7:**
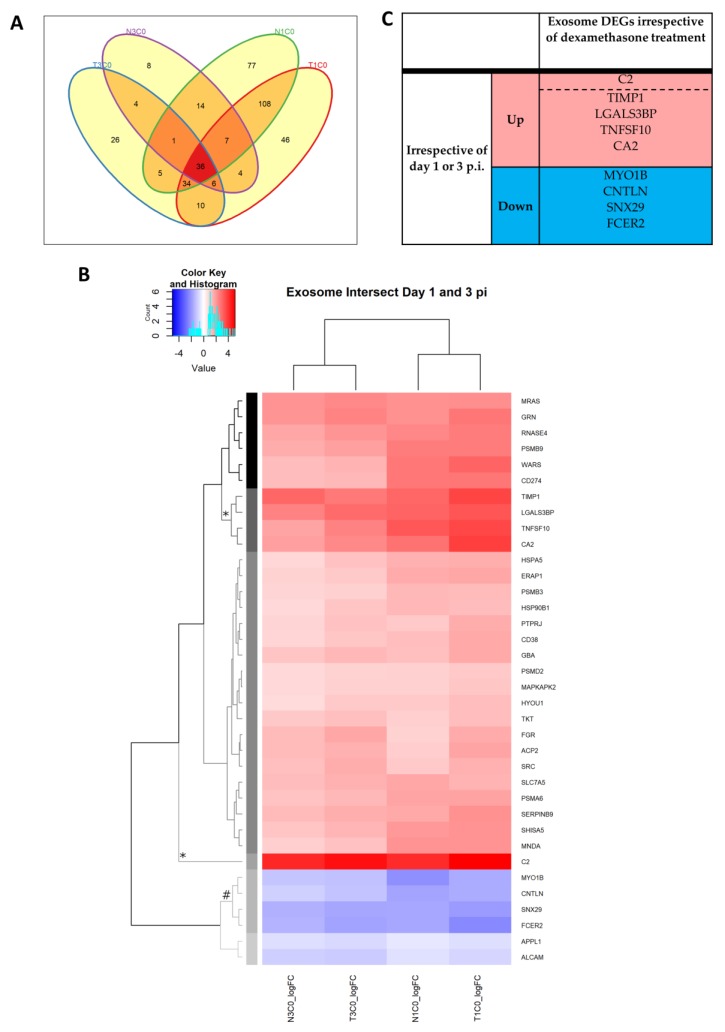
DEGs associated with the GO term “extracellular exosome” on day 1 and 3 p.i. (**A**) Venn diagram showing the overlapping DEGs between the T1C0, T3C0, N1C0 and N3C0 comparisons, as well as the unique DEGs. (**B**) Common DEGs are then analyzed for top up- and downregulated DEGs by hierarchical clustering. The grey scale dendrogram branches and the corresponding sidebar in each heatmap represent the different clusters. Clusters with symbols * and # indicate top up- and downregulated clusters, respectively, summarized in Table 4, Table 5 and Table 6. The blue-red scalebar in the top left corner of each heatmap corresponds to the color scale of the heatmap, indicating the logFC for each gene. The light blue histograms within these scalebars indicate the frequency of genes at each level of logFC. (**C**) Top clusters of exosome DEGs on day 1 and 3 p.i. (summary of Figure 7B).

Belonging in the GO category of cellular compartment, the commonly enriched GO term “extracellular exosome” was analyzed separately to the antiviral GO terms as outlined above (Figure 7). A total of 386 DEGs were associated with this cellular compartment GO term across the day 1 and 3 p.i. comparisons, 36 of which were common across these comparisons (Figure 7A). The top upregulated DEG across all groups was complement 2 (C2; Figure 7B,C). The second cluster of top upregulated DEGs included tissue inhibitor of metalloproteinases 1 (TIMP1), galectin 3 binding protein (LGALS3BP), tumor necrosis factor ligand superfamily member 10 (TNFSF10) and carbonic anhydrase 2 (CA2; Figure 7B,C). Of note, both TIMP1 and TNFSF10 are genes involved in cell proliferation and apoptotic processes, while LGALS3BP has been implicated in NK and lymphokine-activated killer (LAK) cell mediated immune responses. TNFSF10 was also highlighted as one of the top upregulated DEGs on day 3 p.i., irrespective of dexamethasone treatment (Table 6). The top cluster of downregulated DEGs included myosin 1B (MYO1B), centlein (CNTLN), sorting nexin 29 (SNX29) and Fc fragment of immunoglobulin E (IgE) receptor II (FCER2; Figure 7B,C).

#### 2.2.13. Unsupervised Analysis of Gene Expression

**Figure 8:**
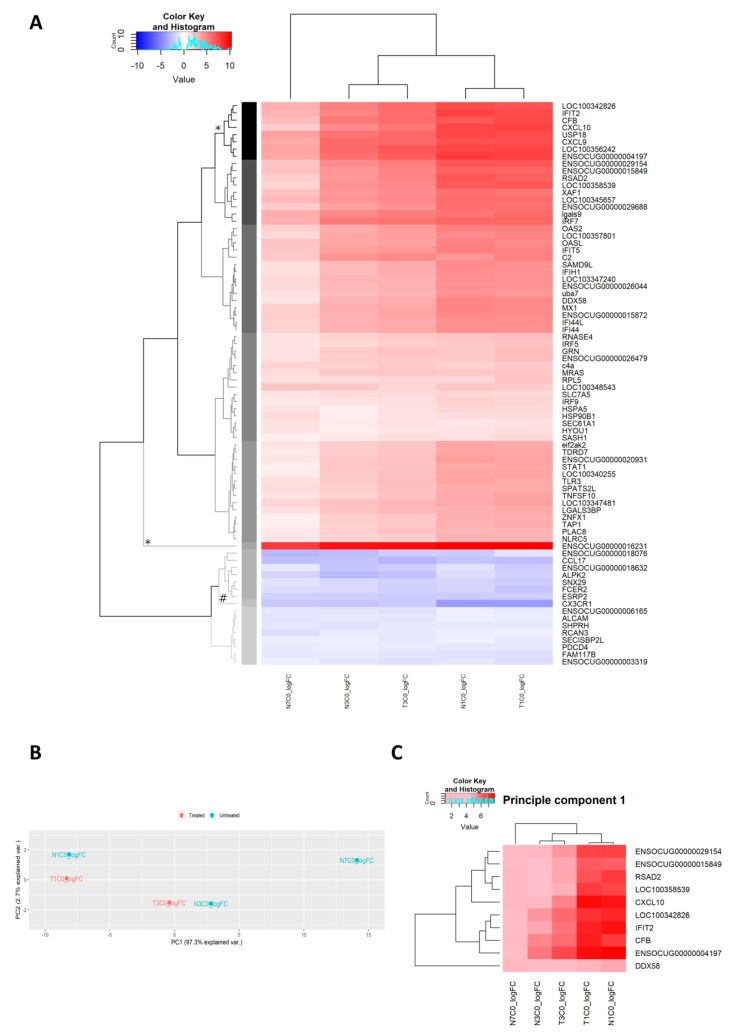
Unsupervised analysis of gene expression. (**A**) Unsupervised hierarchical clustering of DEGs across the five comparisons that yielded DEGs. Note that some genes are annotated with their ensembl gene ID, as these are not available in the DAVID 6.8 database for appropriate conversion to official gene symbols. The grey scale dendrogram branches and the corresponding sidebar in each heatmap represent the different clusters. Clusters with symbols * and # indicate top up- and downregulated clusters, respectively. The blue-red scalebar in the top left corner of each heatmap corresponds to the color scale of the heatmap, indicating the logFC for each gene. The light blue histograms within these scalebars indicate the frequency of genes at each level of logFC. (**B**) Unsupervised PCA shows clustering of the comparisons most prominently by time post-infection. Principle component 1 (PC1) explained 97.3% of the variation of the logFC between the comparisons, whereas PC2 explained 2.7%. (**C**) Heatmap of the top 10 explanatory variables (DEGs) and their logFC.

While gene ontology analyses allowed us to identify groups of genes of interest by using gene functional annotations in a curated database, for example the DAVID 6.8 database in our case, a major limitation to this approach is that genes that are not yet included in the database may not be highlighted as an integral part of the transcriptome. While an extensive unsupervised analysis of gene expression is beyond the scope of this study, we have conducted two simple and common approaches to identifying further genes of interest without a priori assumptions. Firstly, we conducted hierarchical clustering of DEGs from each group analysis based purely on their logFC, followed by principle component analysis (PCA; Figure 8A–C).

For hierarchical clustering, we first subsetted the matrix of DEG logFC to only genes that were differentially expressed across all the pairwise comparisons (i.e. N1C0, N3C0, N7C0, T1C0 and T3C0). This allowed us to identify DEGs that were highly up- or downregulated across the comparisons. A total of 78 genes were differentially expressed across all the comparisons. Of these, a notable top upregulated cluster is identified, containing only one gene, solute carrier family 16 member 5 (SLC16A5; ENSOCUG00000016231), which was highly upregulated across all five comparisons, irrespective of dexamethasone treatment and time post-infection (Figure 8A).

The second top cluster included some genes already identified as key genes across all the comparisons from the GO analyses in Table 4. These included CXCL10 and CXCL9 (Figure 8A). In addition to this, within the second cluster, there were also several genes that have not been previously highlighted in the GO analyses. These included ubiquitin specific peptidase 18 (USP18), complement factor B (CFB), IFIT-1-like protein (LOC100342826), 2’-5’-oligoadenylate synthase-like protein (LOC100356242), and a novel unannotated gene (ENSOCUG00000004197 with closest annotated orthologue being IFIT1B in Gibbon [ENSNLEG00000018636; 68.03% query % identity; 66.88% target % identity], and human orthologue IFIT1B [ENSG00000204010]; Figure 8A). On the other hand, the top downregulated gene cluster contained one gene, the chemokine receptor, CX3CR1, which was most downregulated on day 1 p.i., and to lesser extent, on the subsequent days (Figure 8A).

The subsetted matrix containing 78 DEGs used for hierarchical clustering was also used for PCA, which aimed at identifying DEGs that have the largest effect in explaining the variation of logFC across the different comparisons, N1C0, N3C0, N7CO, T1C0 and T3C0. This allowed identification of DEGs that were the “most different” across the comparisons. The PCA plot in Figure 8B shows that the different comparisons cluster most closely within their corresponding time p.i., with the greatest degree of separation between treatment versus mock-treatment on day 1 p.i. Again, similar to the unsupervised hierarchical clustering, some DEGs already highlighted as important from GO analyses were also present in the top 10 DEGs of the first principal component (PC1), which explained 97.3% of the variation of logFC in the data matrix (Figure 8C and Table 7). These genes included CXCL10, RSAD2 and DDX58 (Table 7). Common to the top clusters from the unsupervised hierarchical clustering, IFIT2, CFB, IFIT1-like protein (LOC100342826) and the novel gene ENSOCUG00000004197 (human orthologue of IFIT1B) were also identified as some of the top ten contributors to the first principal component (Table 7). Unreported in the analyses prior to this, guanylate-binding protein 1 (GBP1; LOC100358539), IFIT3 (ENSOCUG00000029154) and an additional novel gene (ENSOCUG00000015849; Human orthologue: HERC6 [ENSG00000138642]) were identified as important contributing genes to principal component 1 (Table 7).

## 3. Discussion

We have presented data for our studies examining the effect of acute “stress”, in the form of a single systemic injection of the synthetic corticosteroid drug dexamethasone, on viral replication and the antiviral innate immune response following intradermal WNV infection in young rabbits. While virus challenge was done at the time of maximal effect of dexamethasone treatment on immune parameters, the infection, contrary to our hypothesis, resulted in significantly lower viremia in treated rabbits relative to that in the untreated controls. This was likely explained by a different initial innate immune response at the first tissue site of virus replication, the draining lymph node, as demonstrated by the difference in the composition of DEGs within commonly enriched antiviral GO terms in a treatment and time dependent manner.

• Gene ontology

Through the GO analysis, we identified several GO terms with relevance to antiviral immune response that were enriched in each of the pairwise comparisons against control. Enriched biological process and molecular function GO terms “defense response to virus”, “ATP binding”, “negative regulation of viral genome replication”, “positive regulation of JNK cascade”, “double-stranded RNA binding” and “immune response” highlighted the early activation of immune processes in response to the virus challenge. Some of these terms were enriched in a time-dependent manner, in particular, the term “double-stranded RNA binding” was commonly enriched only on day 3 p.i. This coincided with the peak of viral load in the draining lymph node, and therefore, suggests that the expression of molecules for “double-stranded RNA binding” was likely important for restricting virus replication, consistent with the reported literature on WNV-induced immune response [28].

• IFN-I

At the gene level, we pooled DEGs that were associated with enriched GO terms relevant to antiviral immune responses and examined their level of fold change from control. This analysis showed that the IFN response featured prominently, in particular on day 1 p.i., where IFN-β1 was commonly upregulated regardless of dexamethasone treatment. Furthermore, depending on whether the rabbits were treated with dexamethasone or not, different repertoires of IFN-α-21-like and IFN-ω-1-like genes at different loci were expressed on day 1 p.i. While it could be expected that IFN-α and -β would be an important part of the antiviral response, consistent with previous studies on the innate immune response of WNV, flavivirus and viruses in general [28], the finding of concurrent expression of IFN-ω subtypes alongside IFN-α/β is notable. IFN-ω has been reported to possess a comparable level of antiviral activity against flaviviruses such as hepatitis C virus in vitro [29], with increased in vitro antiviral activity when glycosylated, in particular against WNV and other flaviviruses [29]. Our study supports an important role for IFN-ω, perhaps in combination with IFN-α/β, in restricting early WNV replication in rabbits. The role of the various rabbit IFN-α and -ω subtypes in the antiviral action also requires further characterization. Understanding this may help elucidate the complex redundancy of the IFN system available for ancillary support against viral pathogens, when perhaps a particular arm of the immune system may be disturbed by environmental factors such as acute stress.

• IFN-stimulated genes

The IFN system, most notably the type I IFNs, IFN-α and -β, initiates a first-line antiviral response upon viral infection, mediated by the induction of >300 ISGs, leading to the establishment of an antiviral state [30,31]. In the current study, a number of ISGs were highlighted as top upregulated DEGs either in a time/treatment-independent or dependent manner. Regardless of dexamethasone treatment or time post-infection, expression of ISGs of the OAS family, specifically OAS2, OAS3 and OASL, and its downstream RNaseL, were highly upregulated. Generally, ISGs of the OAS family has been well studied and are frequently highlighted in their ability to activate the latent RNase, RNaseL, which in turn degrades viral RNA [32]. Recently, OASL, which lacks the 2’-5’ oligoadenylate catalytic activity, has been described as possessing the ability to boost the antiviral IFN-I response via enhancement of the sensitivity to activate RIG-I [32]. In other words, OASL has been shown to enhance the ability of RIG-I to detect low levels of viral RNA, levels which may be reflective of very early phase of viral replication. In the infected rabbits of the current study, expression of RIG-I (referred to as DDX58 in this manuscript) was upregulated on day 3 p.i., regardless of dexamethasone treatment. Given the overall limited viremia and virus spread in infected rabbits, along with high degree of upregulated expression of OASL and RIG-I, we propose that one of the major mechanisms of early restriction on viral replication in the draining lymph node was mediated by OASL and its enhancement of RIG-I’s sensitivity to low levels of viral RNA. This in turn induced a robust IFN-I response, which was also evident.

Another family of ISGs highly featured in the antiviral response of the rabbits is the IFIT family. IFIT5 was consistently upregulated across all comparisons (Table 4), and from the unsupervised analyses, IFIT1-like, IFIT2, IFIT3 and an unannotated gene with putative IFIT1B function were identified as key genes in the antiviral defense against WNV_NSW2011_. Individually, IFIT1 has been reported to disrupt the interaction of MITA, MAVS and TBK1, which then negatively regulates the cellular antiviral response. IFIT2 interacts with MITA, and induces apoptosis via the mitochondrial pathway that is induced by the innate immune response. IFIT3 bridges TBK1 to MAVS in mitochondria, which synergizes the activation of IRF3 and NF-κB to activate the immune response [33]. We propose that these characterized functions apply also to rabbits, although further investigation into the antiviral mechanisms of the novel IFIT1-like and the putative IFIT1B gene should be conducted.

RSAD2, also known as ‘virus-inhibitory protein, endoplasmic reticulum-associated, interferon-inducible’ (viperin), is another important ISG that was consistently upregulated in infected rabbits in this study. RSAD2/viperin has many diverse functions, antiviral activity being one of these [34], and mediated by multiple pathways, depending on virus species [35,36]. In case of flaviviruses, including WNV, viperin catalyzes conversion of cytidine triphosphate, thereby generating 3’deoxy-3’,4’-didehydro-CTP, which acts as a chain terminator for the RNA-dependent RNA polymerase [35,36]. Viperin also targets the flavivirus NS3 for proteasomal degradation [37] and may interfere with the assembly of the flavivirus capsid [38]. Specifically for WNV, viperin knockout mice were shown to be more susceptible to WNV challenges [39]. The same study also showed tissue and cell specificity for its antiviral function [39].

In a time-specific manner, ISG15 was commonly upregulated on day 1 p.i. regardless of dexamethasone treatment. ISG15, a 15kD protein containing two ubiquitin-like domains [40], forms conjugates with a diverse array of proteins, the specificity of which is in part derived from the temporal expression of the target proteins, in a process referred to as ISGylation. However, there is no evidence that ISGylation leads to proteasome mediated degradation of the target proteins, and thus, the exact molecular mechanism(s) of ISG15 antiviral effect remain inadequately understood and may vary for various viruses and hosts [40]. While ISG15 expression is upregulated in the context of in vivo infection with several flaviviruses [17,41], the antiviral role of ISG15 in WNV infection in vivo has been called into doubt: ISG15^-/-^ mice did not appear to have increased susceptibility to WNV infection despite apparent in vitro antiviral activity of ISG15 towards WNV [40,42]. These conflicting findings need further dissection. Given its time-specific expression in our WNV_NSW2011_-infected rabbits, perhaps ISG15’s role in the overall antiviral defense may also be temporally specific.

Similarly, the expression of a recently characterized ISG, TAP1, was also upregulated in a time-specific manner, except this was observed on day 3 p.i. Traditionally, TAP1 was known for its role as a transporter of proteasome-degradation products to major histocompatibility complex-I (MHC-I) receptors inside the endoplasmic reticulum lumen [43,44]. Hence, it was a regulator of the adaptive immune response to viral infection. However, a study into the antiviral mechanisms against vesicular stomatitis virus infection suggested that TAP1 was an ISG with post-translational processing and degradation of viral protein [44]. Its role in WNV control is poorly understood. Our finding of its common expression on day 3 p.i. highlights a potentially important role and further characterization of this ISG is required.

• IFN-signaling

It is well characterized that the IFN-I response is initiated by the pattern recognition receptors (PRRs) that detect pathogen associated molecular patterns (PAMPs), followed by intracellular signaling. In the current study, consistent with the findings of a robust IFN and ISG response, there was high expression of a variety of PRRs: TLR-3, TMEM173 (also known as STING), NLRC5, DDX58 (RIG-I) and IFIH1 (MDA5). These were upregulated on day 1 and/or 3 p.i. irrespective of dexamethasone treatment. TLR-3 also remained upregulated on day 7 p.i. in the untreated group.

TLR-3 binds double-stranded RNA within endosomes and has been heavily emphasized in its role in initiating an anti-WNV IFN-I response via either the IRF3 or IRF7 signaling pathway [45]. In our previous studies examining the expression of TLR genes in rabbit and equine peripheral blood mononuclear cells (PBMCs) infected with WNV, we observed similar upregulated expression of TLR-3 along with type I IFN and ISGs [46,47]. Therefore, observing upregulated TLR-3 expression in the rabbits of the current experiment further affirmed the importance of this PRR; however, more importantly, that its upregulated expression was maintained even when treated with dexamethasone. This suggests that despite acute stress, the rabbit innate immune system retains its ability to rapidly and persistently deal with WNV infection via TLR-3.

TMEM173 (also known as STING), like TLR-3, is a known initiator of the type I IFN response but unlike TLR-3, it is a PRR that detects cytosolic DNA [48]. Despite this, it has been implicated in the antiviral control of both RNA and DNA viruses [48,49]. The finding of TMEM173/STING transcript upregulation in our current study is consistent with published literature for WNV-induced innate immune response [48]. Using STING knockout mice, it was shown that STING was required for bone-marrow derived macrophages to control WNV replication and to initiate both an effective innate immune response and an appropriate T cell response in vivo in order to restrict WNV-associated neuropathology [48]. Moreover, degradation of STING by flavivirus NS2B3 protease may be one of the immunoevasive mechanisms displayed by these viruses, with the caveat that STING is not subject to cleavage in all species [49]. However, the question why and how a PRR, which senses cytosolic DNA, can be an integral part of the antiviral response to an RNA virus like WNV remains to be elucidated.

NLRC5 was another commonly upregulated gene in the infected rabbits, regardless of dexamethasone treatment. This NOD-like CARD domain-containing intracellular protein has recently been identified as a major histocompatibility complex class I (MHC-I) transactivator [50], but its association with flavivirus infection has apparently not been described. Guo et al. [51] previously showed that respiratory syncytial virus (RSV) infection upregulates MHC-I expression through the induction of NLRC5. In our study, the HLA-I antigen, B7-alpha chain, was concurrently upregulated on day 3 p.i. The link between NLRC5 and MHC-I expression may, therefore, be present in rabbits infected with WNV_NSW2011_, and this interaction may play a key role in virus control, perhaps via the induction of an appropriate cell-mediated adaptive response by cytotoxic T cells. Notably, upregulated expression of PRF1 was observed on day 3 p.i. for untreated rabbits, which may support an important role of the cytotoxic T cell response. NLRC5 may also induce antiviral response against WNV_NSW2011_ via RIG-I signaling pathway, as seen in influenza virus study [52].

In the current study, both DDX58 (RIG-I) and IFIH1 (MDA5) expression were upregulated on day 3 p.i. The importance of DDX58 (RIG-I) was further highlighted by the unsupervised PCA. These two PRRs are well-characterized sensors of dsRNA and mediate antiviral IFN-I response via IRF3 and 7 [50]. However, it is notable that the expression of the regulator of both the RIG-I and MDA5 pathway, DHX58 (also known as LGP2), was also upregulated at the same time. This suggests that a fine-tuning regulatory mechanism within the RIG-I and MDA5 pathways is present in the rabbit’s antiviral response against WNV_NSW2011_.

• Adaptive response on day 3 p.i.

Throughout the analyses of day 1 and 3 p.i., there were indications that adaptive immune response involving specific T cell subsets may be integral to the restricting WNV infection. As mentioned already, upregulated expression of PRF1 on day 3 p.i. of untreated rabbits hinted at a cytotoxic T cell or NK cell response. The early upregulation of IL23A, as well as IFNγ on day 3 p.i. in dexamethasone treated rabbits suggest a CD4^+^ T lymphocytic response (helper 17 and helper 1, respectively) was preferred in this group. The kinetics of leukocytes through the lymph node was also likely driven by the temporal pattern of chemokine ligand and receptor expression [53].

• Chemokines ligand and receptor expression

Mobilization of effector cells to sites of active infection is driven by, amongst others, chemokines. CXC type chemokines that lack the ELR-sequence, including CXCL9 and CXCL10, are potent attractants for activated T and B lymphocytes, monocytes, dendritic cells and NK cells [53,54]. Both of these chemokines bind to the chemokine receptor CXCR3, the signaling through which has been shown to be very important in many viral infections [54,55,56,57]. We previously demonstrated upregulation of CXCL10 in brain and draining lymph node following WNV infection in rabbits [20,21] and this was validated for the draining lymph node in this study in both dexamethasone treated and untreated rabbits. We also demonstrated equally potent upregulation of CXCL9 in a similar time- and treatment-independent manner (Figure 6B, Table 4). CXCL11 was also highlighted as a top upregulated gene specifically on day 3 p.i. (Figure 6D), emphasizing the importance of CXC type chemokine response to restricting WNV replication in vivo.

Despite the upregulation of the CXC type chemokines, it is noteworthy that genes encoding other mononuclear leukocyte chemokines like CCL17 (irrespective of dexamethasone treatment) and CCL22 (specific to dexamethasone treated rabbits) were prominently downregulated, alongside their receptors CCR8 and CCR4, respectively. This potentially hints at a complex regulation of chemoattraction of specific mononuclear leukocytic subsets with specific activation state to the lymph node in order to achieve virus control.

• Extracellular exosome

Alongside the use of biological process and molecular function GO terms, we included terms in the category of cellular compartment for the GO analyses. Notably, the “extracellular exosome” compartment was repeatedly enriched across all comparisons. Analysis into the composition of the top DEGs within this term revealed that the genes encoding C2, as well as TIMP1, LGAL3BP, TNFSF10 and CA2, were highly upregulated on day 1 and 3 p.i. regardless of dexamethasone treatment. Upregulation of C2 was the most consistently pronounced across these comparisons. This suggests an important role of complement in the antiviral response against WNV, potentially via extracellular exosomes. TIMP1 and TNFSF10 have been implicated in the control of cell proliferation and apoptotic processes [58,59]. These molecules in exosomes may potentially drive the formation of germinal centers in the lymph nodes for achieving a competent antibody response, which was apparent in these rabbits (Section 2.2.4). This exciting field of exosome-mediated antiviral mechanisms should be further investigated.

• Unsupervised analyses identified genes with little-known antiviral activity

Apart from validating the importance of antiviral genes, such as the ISGs, DDX58 and the chemokines CXCL9 and 10, the unsupervised analyses also highlighted genes with little-known antiviral activity. Notably, SLC16A5 was consistently upregulated to the highest extent across all groups, regardless of dexamethasone treatment or time post-infection. This protein, also known as monocarboxylate transporter 6, aids in the transport of monocarboxylates (e.g., lactate, pyruvate), ketone bodies and branched-chain oxo acids across cell plasma membrane [60]. Nothing is known about its association with antiviral activity.

Another gene identified as important by our unsupervised approach was the unannotated gene with putative HERC6 function. HERC6 is a ubiquitin ligase and has been implicated in MHC class I mediated antigen processing [61]. Further characterization of this novel rabbit gene with putative HERC6 function is warranted.

• Corticosteroids and immunosuppression

The current study showed that the altered immune function associated with a single bolus injection of dexamethasone was insufficient for induction of severe disease upon WNV infection. This challenges the perception that corticosteroid administration or acute stress will compromise immune function to the point of increasing susceptibility to infectious diseases. It is evident from our study that the action of corticosteroids on the immune function is complex and the outcome of its use in the context of WNV infected patients may be dose-, frequency- and timing-dependent. Further dissection into these aspects by varying the corticosteroid dose, frequency and time of administration in relation to WNV infection is required.

In conclusion, acute stress as modeled by a single dose dexamethasone injection in rabbits does not induce a more severe disease phenotype when infected with WNV_NSW2011_, contrary to our initial hypothesis. We have shown that despite acute stress, WNV-infected rabbits retained their ability to activate an early antiviral response, primarily via type I IFNs and in particular IFN-β1, IFN-α-21-like and IFN-ω-1-like subtypes, as well as the induction of a range of ISGs (OASs, IFITs, RSAD2/viperin, and ISG15). The involvement of IFN-ω in the anti-WNV immune response is a unique highlight of the rabbit model. We propose that this robust IFN-I response was likely initiated and sustained by ongoing expression of TLR-3 and TMEM173/STING, with DHX58-regulated IFIH1 (MDA5)- and DDX58 (RIG-I)-mediated signaling modulating the IFN-I response. Despite these commonalities, a lower viremia and faster return of the draining lymph node transcriptome to baseline levels were observed in the dexamethasone treated rabbits. This highlights a slightly different immune response, as supported by the expression of the different repertoire of IFN-α-21-like and IFN-ω-1-like subtypes on day 1 p.i., which in turn may have driven the different chemokine response between treated and untreated rabbits on day 3 p.i. Future studies in this model should be extended to transcriptomic analysis of lymphoid organs distal to the draining lymph node, e.g., spleen and other lymph nodes, with a comparison of the transcripts in the draining versus distal lymphoid organs. We have also identified few genes upregulated to high magnitude, but with little known association with WNV restriction. These included SLC16A5 and an unannotated gene with putative HERC6 function. We have also identified another unannotated gene with putative IFIT1B function, paving potential new research into the profile of novel ISGs in rabbits. The acute stress model as presented in the current study therefore shows that rabbits are capable of restricting WNV replication and dissemination by a robust innate immune system that can retain its antiviral function despite environmental challenges such as stress.

## 4. Materials and Methods

### 4.1. Virus

The WNV_NSW2011_ stock used in this study is the same as that previously used in the initial characterization study of the rabbit WNV-challenge model [20]. The stock has the same passage history and same titer as previously described [20]. Briefly, the WNV_NSW2011_ stock originally derived from a brain isolate of a horse during the 2011 outbreak, propagated in C6/36 cells, then passaged once in Vero cells, and twice further in C6/36 cells, before being used in the current study [3,4,20].

### 4.2. Animals and Experimental Design

All experiments were performed at the University of Queensland, Gatton Campus, in accordance with the ethical standards of The Australian Code for the Care and Use of Animals for Scientific Purposes and were approved by the University of Queensland Production Animal Ethics Committee before commencement of the study (Permit No. SVS/555/17). Rabbits used in this study were 4–5 weeks old NZWRs, sourced from Nanowie (Victoria, Australia) and the University of Queensland Biological Resources (UQBR). The study had two phases: In an initial pilot experiment, four weanling NZWRs were injected with 6.5 mg/kg dexamethasone sodium phosphate (Dexapent, 5 mg/mL; Troy Laboratories, Glendenning, NSW, Australia) intramuscularly in the semimembranosus muscle, split into two doses of 0.3–0.4 mL between the left and right hind legs. This dose of dexamethasone was based on that used by Jeklova et al. [26], but modified to combine the multiple injections to a single bolus injection. The effects of this modified dexamethasone dosing regime on immune parameters were assessed, and this subsequently informed the optimal day post-dexamethasone treatment for WNV inoculation. Two age-matched NZWRs were used as mock-treatment control animals for comparison. Post-dexamethasone treatment, all rabbits were monitored twice daily and bled daily to assess hematological changes and blood cytokine transcript profile. Two treated and one control rabbit were culled on day 3 and 7 post-treatment for tissue collection for histopathology and downstream molecular assays. For the euthanasia, all animals were sedated with xylazine (Xylazil, 20 mg/mL, Troy Laboratories, Glendenning, NSW, Australia), followed by an overdose of pentobarbital (Lethabarb, Troy Laboratories), as previously described [20].

This pilot experiment suggested the optimal day post-dexamethasone injection for WNV inoculation was day 3 pdt (see Section 2.1). For the challenge experiment, nine NZWRs were treated with dexamethasone, as described above, 3 days prior to challenge with 10^5^ TCID_50_ of WNV_NSW2011_ in 50 µL media intradermally in the left footpad, as per our previous studies [20]. Seven rabbits were used as mock-treatment controls, receiving a volume of PBS similar to that of the dexamethasone intramuscularly. These rabbits were challenged with the same dose of WNV_NSW2011_ by the same route as the dexamethasone-treated group. The animals were assessed clinically twice daily and bled once daily. The termination schedule was as indicated in Table 8.

At termination, a complete post-mortem examination was performed on each rabbit with collection of a range of organs and tissues for histopathology and downstream virological and molecular assays.

In order to reduce the amount of environmental variability that may influence the stress level of the rabbits, the ambient temperature and day/night cycle were maintained constant throughout the trial, and husbandry practices and blood sampling were performed at approximately the same time daily. The two animal trials were also performed in the same room in the same animal holding facility under the same conditions, thus justifying the use of the tissues from untreated uninfected rabbits 2001 and 2002 from the pilot trial as baseline controls for the differential gene expression analysis between dexamethasone-treated and untreated WNV-infected rabbits. The use of these controls was also required, as the number of animals used in the second trial reached maximum capacity for the animal room (at n = 16), and no additional clean animals could be included in the trial as baseline controls.

### 4.3. Hematology

Immediately after blood sample collection into EDTA tubes (Multivette^®^ 600, Sarstedt Ag & Co.), 3 µL of whole blood was used to prepare a routine blood smear on single frosted glass slides. Three blood smears were prepared from each animal at each bleed. After air drying by waving slides in the air for few seconds, smears were fixed immediately in 100% methanol for 5 min. Smears were then air dried, before staining with Wright-Giemsa stain in a Siemens Hematek^®^ 1000 autostainer (Siemens Corp., Tarrytown, NY, USA). All blood smears were examined using a Nikon Eclipse *Ci* microscope at 10× or 20× objectives for manual cell counting and 20× to 40× objectives for differential counts.

### 4.4. Histopathology

Tissue samples were harvested immediately after euthanasia and fixed in 10% neutral buffered formalin solution for 48 h before being transferred into 70% ethanol for storage until trimming and routine processing for paraffin embedding. 4 µm thick sections were stained with hematoxylin and eosin and examined on a Nikon Eclipse 51 E microscope. Digital microphotographs were taken using a Nikon DS-Fi1 camera with a DS-U2 unit and NIS elements F 4.60 software. Images are reproduced without manipulations other than cropping and adjustment of light intensity.

### 4.5. Immunohistochemistry

Serial sections (4 µm) were cut from formaldehyde-fixed, paraffin-embedded samples from the popliteal lymph nodes, spleen, thymus, mesenteric lymph node, the virus injection site (left footpad) and brain and subjected to IHC-labeling as previously described in detail [17,21]. Briefly, following deparaffinization, antigen retrieval and several blocking steps, the sections were incubated with primary antibody (details listed in Table 9) followed by visualization of the binding using the Envision kit from DAKO, specific for either mouse or rabbit immunoglobulin as appropriate. The sections were counterstained with Meyer’s hematoxylin and examined on a Nikon Eclipse 51 E microscope. A semi-quantitative score was applied that took into account both number of positive cells and labeling intensity for each marker [17]. Digital micrographs were generated as described above.

### 4.6. Cell culture and Virus Isolation

Virus titration was performed by both tissue culture infective dose 50% end-point (TCID_50_) and plaque assay on serum (viremia) and tissues (10% tissue homogenate) using monolayers of Vero cells (African green monkey kidney cells) as previously described in detail [20].

### 4.7. RNA Extraction and Viral Transcript Quantitation

Half of each draining lymph node harvested during post-mortem was submerged into liquid nitrogen immediately after harvest in order to preserve RNA integrity. RNA extraction was performed by first homogenizing the tissue in 1 mL of Qiazol (Qiagen, Chadstone Centre, Vic, Australia), followed by DNase I digestion in solution and RNA cleanup with RNeasy columns, as previously described [20,21]. Prior to homogenization in Qiazol, the tissue was weighed, in order to allow expression of virus quantitation per gram of input tissue. WNV RNA in samples was quantified by first reverse transcription into cDNA using the qScript first strand synthesis kit (Quanta Bioscience, Gaithersburg, MD, USA) and then qPCR using the Rotorgene SYBR green kit (Qiagen) [20,21]. Using 10-fold dilution series of cDNA derived from RNA extracted from WNV stock with known TCID_50_ titer, a standard curve was generated along each qPCR run for absolute quantitation (R^2^ of 0.9386 and a limit of detection of 1.3 TCID_50_). RNA extraction from whole blood was achieved by lysing up to 100 uL of whole blood in 1mL of Qiazol by vortexing. The downstream processing and cytokine transcript qRT-PCR was as described above, using primers as previously described [21].

### 4.8. RNAseq

RNA quality was initially screened by Agilent 2100 Bioanalyzer. Samples with a RIN score of greater than or equal to 4.9 were used for RNA-Seq library preparation, using the Illumina TruSeq Stranded Total RNA LT (Ribo-Zero Gold) Sample Prep Kit (Illumina, RS-122-2301/RS-122-2302), according to the standard manufacturer’s protocol (Illumina, 15031048 Rev. E October 2013). Briefly, to enrich for mRNA, 1 µg of total RNA was depleted of rRNA using Ribo-Zero Gold. The enriched mRNA was then subjected to a heat fragmentation step aimed at producing fragments between 130–290 bp (average 185 bp). This step was adjusted to 7 minutes in order to account for the generally low RIN score. cDNA was synthesized from the fragmented RNA using SuperScript II Reverse Transcriptase (Invitrogen, 18064014) and random primers. The resulting cDNA was converted into dsDNA in the presence of dUTP to prevent subsequent amplification of the second strand and thus maintaining the ‘strandedness’ of the library. Following 3’ adenylation and adaptor ligation, libraries were subjected to 15 cycles of PCR to produce libraries ready for sequencing. The libraries were quantified on the Perkin Elmer LabChip GX with the DNA High Sensitivity Reagent kit (Perkin Elmer, CLS760672). Libraries were pooled in equimolar ratios, and the pool was quantified by qPCR using the KAPA Library Quantification Kit—Illumina/Universal (KAPA Biosystems, KK4824) in combination with the Life Technologies Viia 7 real time PCR instrument. Sequencing was performed using the Illumina NextSeq500 (NextSeq control software v2.1.0/Real Time Analysis v2.4.11). The library pool was diluted and denatured according to the standard NextSeq protocol (Document # 15048776 v02), and sequenced to generate paired-end 76 bp reads using a 150 cycle NextSeq500/550 High Output reagent Kit v2 (illumina, FC-404-2002). After sequencing, fastq files were generated using bcl2fastq2 (v2.18.0). Library preparation and sequencing was performed at the Institute for Molecular Bioscience Sequencing Facility (The University of Queensland). Note that RNA samples extracted from the two uninfected control rabbits were spiked with RNA extracted from the WNV_NSW2011_ inoculum used for the challenge study. This was performed for the possibility to investigate viral genomic dynamics in the draining lymph node in the future.

### 4.9. Differential Gene Expression Analysis

All RNAseq data preparation and differential gene expression analyses were performed using tools within the web-based Galaxy Australia platform [62]. Paired-end reads for each sample, including controls, were mapped to the rabbit genome (OryCun2.0, GenBank assembly ID: GCA_000003625.1) using the gapped alignment tool TopHat (Galaxy version 2.1.1). A matrix of gene read counts for each sample was generated using the tool, “SAM/BAM to count matrix using HTseq code” (Version 0.5), with read quality filter set at MAPQ score of 5. Three R-based differential gene expression analysis packages under the Galaxy tool “Differential _Count models using BioConductor packages” (Galaxy Version 0.28) were used: 1) EdgeR, 2) DEseq2 and 3) Voom-limma. DEGs from each package were determined by an adjusted *p*-value ≤ 0.05. Only genes that were commonly differentially expressed across all three tools were then used for downstream GO analyses using the web tool, DAVID 6.8. Enriched GO terms and their associated genes were analyzed and graphically visualized using the R packages, GOplot (version 1.0.2) and Vennerable (Version 3.1.0.9000). Heatmaps were generated using heatmap.2 from the R package, gplots (Version 3.0.1.1), with rows ordered based on dendrogram and hierarchical clustering using the hclust function (R) under “complete” mode. Principle component analysis was conducted using the R package, mixOmics (version 6.7.0).

### 4.10. Serology

Quantification of anti-WNV specific antibodies was performed by two methods: blocking enzyme-linked immunosorbent assay (blocking-ELISA) [63] and microneutralization assay [64]. The blocking ELISA protocol has been described extensively in previous publications [2,63,64]. The current assay used lysate from C6/36 cells infected with KUNV strain MRM61C as the coating antigen. Monoclonal antibodies used for these competitive assays were either an anti-flavivirus envelope monoclonal antibody, 6B6C-1, or an anti-WNV NS1 specific monoclonal antibody, 3.1112G [63,64]. A cut-off of 30% inhibition was used to determine positive samples [63].

## Figures and Tables

**Table 1:** Immunohistochemical detection of WNV infected cells in various tissues.

Pre-Treat	Day p.i.	Rabbit ID	L. Ln Popl	R. Ln Popl	Spleen	Thymus	Ln. Mes	Left Footpad	Right Footpad	Brain	Liver
Dex	1	2101	0	0	0	0	0	0	0	ND	0
		2102	+	0	0	0	0	0	0	ND	0
		2103	+	0	0	0	0	0	0	ND	0
	3	2104	+	0	0	0	0	0	0	ND	ND
		2105	++	+++	+	0	++	0	0	ND	ND
		2106	+	0	+	0	0	0	0	ND	ND
	7	2107	0	0	0	0	0	0	0	0	ND
		2108	0	0	0	0	0	0	0	ND	ND
		2109	0	0	0	0	0	0	0	ND	ND
PBS	1	2110	+++	0	0	0	0	0	0	ND	0
		2111	0	0	0	0	0	0	0	0	ND
		2112	++	0	0	0	0	0	0	ND	ND
	3	2115	+	0	0	0	0	0	0	ND	ND
		2116	+	0	0	0	0	0	0	ND	0
	7	2113	0	0	0	0	0	0	0	ND	ND
		2114	0	0	0	0	0	0	0	ND	0

Dex, dexamethasone-treated group; PBS, mock-treated group; L. Ln Popl, left popliteal lymph node; R. Ln Popl, right popliteal lymph node; Ln. Mes, mesenteric lymph node; 0, no antigen detected; +, low antigen load; ++, moderate antigen load; +++, heavy antigen load.

**Table 2:** Summary of group name abbreviations used for RNAseq data analyses.

Group Abbreviation	Day Post-Infection	WNV_NSW2011_ Infected	Dexamethasone Treated	Rabbit IDs
C0	N/A	N	N	2001 and 2002 *
N1	1	Y	N	2110, 2111, and 2112
N3	3	Y	N	2115 and 2116
N7	7	Y	N	2113 and 2114
T1	1	Y	Y	2101 and 2102 ^#^
T3	3	Y	Y	2104, 2105, and 2106
T7	7	Y	Y	2107, 2108, and 2109

N/A, not applicable. * Control rabbits 2001 and 2002 were mock-treated controls from the pilot experiment. ^#^ Rabbit 2103 was excluded due to a low RIN (RNA integrity number) score.

**Table 3:** Summary of selected enriched gene ontology (GO) terms and the time post-infection at which they are enriched. Terms are selected on the criteria that they are relevant to antiviral processes and are enriched on two or more occasions.

Selected Enriched GO Terms	Treated		Mock-Treated		
	Day 1	Day 3	Day 1	Day 3	Day 7
defense response to virus (GO:0051607, BP)	X	X	X	X	X
extracellular exosome (GO:0070062, CC)	X	X	X	X	X
ATP binding (GO:0005524, MF)	X	X		X	
negative regulation of viral genome replication (GO:0045071, BP)	X		X	X	
positive regulation of JNK cascade (GO:0046330, BP)	X		X		
double-stranded RNA binding (GO:0003725, MF)		X		X	
immune response (GO:0006955, BP)	X	X		X	

Days indicated refer to days p.i.; shaded squares containing a central cross indicate the time p.i. where the GO term is enriched. Number in brackets indicate the GO term ID. This is followed by their category: BP, biological process; CC, cellular component; MF, molecular function.

**Table 4:** Top upregulated antiviral DEGs irrespective of dexamethasone treatment on day 1 and 3 p.i. (summary of Figure 6B).

Antiviral DEGs Irrespective of Dexamethasone Treatment
CXCL9 CXCL10 RSAD2 (viperin)
OASL OAS2 IFIT5
TMEM173 (STING) IRF1 NLRC5 TLR3 OAS3 RNaseL

Note: only the top three clusters of upregulated DEGs are summarized in this table (hierarchical clustering was performed based on the log-fold change of the genes). Clusters are demarcated by dashed borders.

**Table 5:** Top clusters of antiviral DEGs on day 1 p.i. (summary of Figure 6C,E,F).

Day 1 p.i.	Antiviral Genes Affected by Dexamethasone Treatment		Antiviral DEGs Irrespective of Dexamethasone Treatment
	Expressed in Treated Group	Expressed in Mock-Treated Group	
**Up**	LOC100355669 (IFN-α-21-like)	LOC100353640 (IFN-ω-1-like)	LOC100354397 (IFN-ω-1-like)
	LOC100354910 (IFN-ω-1-like)	LOC100353137 (IFN-ω-1-like)	IFNβ1
	LOC100355421 (IFN-ω-1-like)	LOC100357194 (IFN-α-21-like)	LOC100354654 (IFN-α-21-like)
			LOC100358223 (IFN-ω-1-like)
			LOC100357708 (IFN-α-21-like)
			ISG15
	LOC100346553 (multidrug resistance-associated protein 1)	LOC100353888 (IFN-ω-1-like)	NOX1
	CCR1	GADD45G	IL1β
	IL23A		
	CCL2		
**Down**	PDK4	—	GADD45A
	MYO6		CCL21
	FGFR1		RASGRP1
	TGFBR3		TPD52L1
	KATNAL1		
	CNNM2		
	ABCA8		
	PLCB1		
	DYNC2H1		
	ABCC5		
	NPR1		
	ACTA2		
	EPHB3		
	LOC100358984 (vascular endothelial growth factor receptor kdr-like)		

Note: only top 1–2 clusters of upregulated or downregulated DEGs are summarized in this table (hierarchical clustering was performed based on the log-fold change of the genes). Clusters are demarcated by dashed borders.

**Table 6:** Top clusters of antiviral DEGs on day 3 p.i. (summary of Figure 6D,G,H).

Day 3 p.i.	Antiviral Genes Affected by Dexamethasone Treatment		Antiviral DEGs Irrespective of Dexamethasone Treatment
	Expressed in Treated Group	Expressed in Mock-Treated Group	
**Up**	IFNγ	LOC100348075(tubulin alpha-1B chain-like)	CXCL11
		HSPA8	
		PRF1	
	CCR2	LOC100358336 (actin-related protein 3-like)	IFIH1 (MDA5)
	LOC100349255 (lymphotactin)	FKBP4	DDX58 (RIG-I)
	LOC100349247 (HLA class I histocompatibility antigen, B-7 alpha chain)	ABCA1	DHX58 (LGP2)
			HK3
			TNFSF10 (TRAIL)
			TAP1
**Down**	CCL22	TOP2B	CCR8
	CCR4	DGKH	ALPK2
	LOC100350168 (HLA class II histocompatibility antigen, DRB1-4 beta chain)		CCL17
	PRKRA		KIF5C
	PLK2		

Note: only the top 1-2 clusters of upregulated or downregulated DEGs are summarized in this table (hierarchical clustering was performed based on the log-fold change of the genes). Clusters are demarcated by dashed borders.

**Table 7:** Top 10 DEGs contributing to PC1 and their weights in the loading vector for PC1.

Gene ID	Weights	Gene Description
CXCL10	−0.2492985	C-X-C motif chemokine ligand 10
LOC100358539	−0.2233737	guanylate-binding protein 1
RSAD2	−0.2079025	radical S-adenosyl methionine domain containing 2
IFIT2	−0.1999001	interferon induced protein with tetratricopeptide repeats 2
ENSOCUG00000029154	−0.191678	interferon induced protein with tetratricopeptide repeats 3
CFB	−0.1915019	complement factor B
ENSOCUG00000004197	−0.1883572	novel gene (Human orthologue: IFIT1B [ENSG00000204010])
LOC100342826	−0.1697197	interferon-induced protein with tetratricopeptide repeats 1-like
ENSOCUG00000015849	−0.1678594	novel gene (Human orthologue: HERC6 [ENSG00000138642])
DDX58	−0.1648038	DExD/H-box helicase 58

**Table 8:** Termination schedule for the WNV challenge experiment.

	Day 1 p.i.	Day 3 p.i.	Day 7 p.i.
Dexamethasone-treated	n = 3 (ID: 2101-3)	n = 3 (ID: 2104-6)	n = 3 (ID: 2107-9)
Mock-treated	n = 3 (ID: 2110-2)	n = 2 (ID: 2115-6)	n = 2 (ID: 2113-4)

**Table 9:** List of antibodies used for IHC and conditions applied.

Antibody	Source	Specificity	Antigen Retrieval	1^o^ Ab Incubation	2^o^ Ab (Envision Kit)
Anti-flavivirus NS1 (clone 4G4)	SCMB (UQ)	Flavivirus Non-Structural 1 (NS1) protein	EDTA, pH9	2 hours r.t.	Anti-Mouse Anti-rabbit
Anti-human myeloid/histiocyte antigen (clone Mac 387)	DAKO	Myeloid lineage cells	Proteinase K		
CD 3 (clone F7.2.38)	DAKO	T lymphocytes	EDTA, pH9		
CD 79a (clone HM57)	DAKO	B lymphocytes	Citrate, pH6	Overnight, 4 °C	
Ki67	Abcam	Proliferation marker	EDTA, pH9	1–2 hours r.t.	
Activated caspase-3	Abcam	Apoptosis marker	EDTA, pH9	Overnight, 4 °C	

## Supplementary Materials

The following are available online at https://www.mdpi.com/2076-0817/8/4/195/s1, Figure S1: Effect of bolus administration of dexamethasone on hematologic profile in uninfected rabbits. Figure S2: Effect of dexamethasone treatment on mRNA expression for a select set of cytokines in whole blood. Figure S3: Effect of bolus administration of dexamethasone on thymic morphology in young rabbits. Figure S4: Enriched GO terms from the pairwise comparisons. Figure S5: DEGs associated with GO terms relevant to antiviral immune responses on day 7 p.i. Table S1: Top clusters of antiviral DEGs on day 7 p.i.

## References

[1] Centers for Disease Control & Prevention (CDC) West Nile virus final cumulative maps & data for 1999–2018. https://www.cdc.gov/westnile/statsmaps/cumMapsData.html.

[2] Prow N.A., Hewlett E.K., Faddy H.M., Coiacetto F., Wang W., Cox T., Hall R.A., Bielefeldt-Ohmann H. (2014). The Australian public is still vulnerable to emerging virulent strains of WNV. Front. Public Health..

[3] Prow N.A., Edmonds J.H., William D.T., Setoh Y.X., Bielefeldt-Ohmann H., Suen W.W., Hobson-Peters J., Van den Hurk A.F., Pyke A.F., Hall-Mendelin S. (2016). Virulence and evolution of West Nile virus, Australia, 1960–2012. Emerg. Infect. Dis..

[4] Frost M.J., Zhang J., Edmonds J.H., Prow N.A., Gu X., Davis R., Hornitzky C., Arzey K.E., Finlaison D., Hick P. (2012). Characterization of virulent WNV Kunjin strain, Australia, 2011. Emerg. Infect. Dis..

[5] Lindsey N.P., Staples J.E., Lehman J.A., Fischer M. (2010). Surveillance for human WNV disease—United States, 1999–2008. MMWR Surveill. Summ..

[6] Sejvar J.J., Lindsay N.P., Campbell G.L. (2011). Primary causes of death in reported cases of fatal West Nile fever, United States, 2002–2006. Vector Borne Zoonotic Dis..

[7] Jean C.M., Honarmand S., Louie J.K., Glaser C.A. (2007). Risk factors for West Nile virus neuroinvasive disease, California, 2005. Emerg. Infect. Dis..

[8] Lindsey N.P., Staples J.E., Lehman J.A., Fischer M. (2012). Medical risk factors for severe West Nile virus disease, United States, 2008–2010. Am. J. Trop. Med. Hyg..

[9] Grubaugh N.D., Massey A., Shives K.D., Stenglein M.D., Ebel G.D., Beckham J.D. (2015). West Nile Virus Population structure, injury, and interferon-stimulated gene expression in the brain from a fatal case of encephalitis. Open Forum Infect. Dis..

[10] Suen W.W., Prow N.A., Hall R.A., Bielefeldt-Ohmann H. (2014). Mechanism of West Nile virus neuroinvasion: A critical appraisal. Viruses.

[11] Gause K.T., Wheatley A.K., Cui J., Yan Y., Kent S.J., Caruso F. (2017). Immunological principles guiding the rational design of particles for vaccine delivery. ACS Nano.

[12] McClain M.T., Henao R., Williams J., Nicholson B., Veldman T., Hudson L., Tsalik E.L., Lambkin-Williams R., Gilbert A., Mann A. (2016). Differential evolution of peripheral cytokine levels in symptomatic and asymptomatic responses to experimental influenza virus challenge. Clin. Exp. Immunol..

[13] Sun Y., Lopez C.B. (2017). The innate immune response to RSV: Advances in our understanding of critical viral & host factors. Vaccine.

[14] Werner J.M., Heller T., Gordon A.M., Sheets A., Sherker A.H., Kessler E., Bean K.S., Stevens M., Schmitt J., Rehermann B. (2013). Innate immune responses in hepatitis C virus-exposed healthcare workers who do not develop acute infection. Hepatology.

[15] Prow N.A., Setoh Y.X., Biron R.M., Sester D.P., Kim K.S., Hobson-Peters J., Hall R.A., Bielefeldt-Ohmann H. (2014). The West Nile virus-like flavivirus Koutango is highly virulent in mice due to delayed viral clearance and the induction of a poor neutralizing antibody response. J. Virol..

[16] Suen W.W., Prow N.A., Setoh Y.X., Hall R.A., Bielefeldt-Ohmann H. (2016). End-point disease investigation for virus strains of intermediate virulence as illustrated by flavivirus infections. J. Gen. Virol..

[17] Bielefeldt-Ohmann H., Bosco-Lauth A., Hartwig A.E., Uddin M.J., Barcelon J., Suen W.W., Wang W., Hall R.A., Bowen R.A. (2017). Characterization of non-lethal West Nile Virus (WNV) infection in horses: Subclinical pathology and innate immune response. Microb. Pathog..

[18] Graham J.B., Thomas S., Swarts J., McMillan A.A., Ferris M.T., Suthar M.S., Treuting P.M., Ireton R., Gale M., Lund J.M. (2015). Genetic diversity in the Collaborative Cross model recapitulates human WNV disease outcomes. mBio.

[19] Seok J., Warren H.S., Cuenca A.G., Mindrinos M.N., Baker H.V., Xu W., Richards D.R., McDonald-Smith G.P., Gao H., Hennessy L. (2013). Genomic responses in mouse models poorly mimic human inflammatory diseases. Proc. Natl. Acad. Sci. USA.

[20] Suen W.W., Uddin M.J., Wang W., Brown V., Adney D.R., Broad N., Prow N.A., Bowen R.A., Hall R.A., Bielefeldt-Ohmann H. (2015). Experimental West Nile virus infection in rabbits: an alternative model for studying induction of disease and virus control. Pathogens.

[21] Suen W.W., Uddin M.J., Prow N.A., Bowen R.A., Hall R.A., Bielefeldt-Ohmann H. (2016). Tissue-specific transcription profile of cytokine and chemokine genes associated with flavivirus control and non-lethal neuropathogenesis in rabbits. Virology.

[22] Kirschman L.J., Crespi E.J., Warne R.W. (2018). Critical disease windows shaped by stress exposure alter allocation trade-offs between development and immunity. J. Anim. Ecol..

[23] Aljebab F., Choonara I., Conroy S. (2016). Systematic review of the toxicity of short-course oral corticosteroids in children. Arch. Dis. Child..

[24] Singanayagam A., Glanville N., Girkin J.L., Ching Y.M., Marcellini A., Porter J.D., Toussaint M., Walton R.P., Finney L.J., Aniscenko J. (2018). Corticosteroid suppression of antiviral immunity increases bacterial loads and mucus production in COPD exacerbations. Nat. Commun..

[25] Alfakeekh K., Azar M., Sowailmi B.A., Alsulaiman S., Makdob S.A., Omair A., Albanyan E., Bawazeer M.S. (2019). Immunosuppressive burden and risk factors of infection in primary childhood nephrotic syndrome. J. Infect. Public Health.

[26] Jeklova E., Leva L., Jaglic Z., Faldyna M. (2008). Dexamethasone-induced immunosuppression: A rabbit model. Vet. Immunol. Immunopathol..

[27] Nakagawa M., Terashima T., D’yachkova Y., Bondy G.P., Hogg J.C., Van Eeden S.F. (1998). Glucocorticoid-induced granulocytosis: Contribution of marrow release and demargination of intravascular granulocytes. Circulation.

[28] Lazear H.M., Diamond M.S. (2015). New insights into innate immune restriction of West Nile virus infection. Curr. Opin. Virol..

[29] Buckwold V.E., Wei J., Huang Z., Huang C., Nalca A., Wells J., Russell J., Collins B., Ptak R., Lang W. (2007). Antiviral activity of CHO-SS cell-derived human omega interferon and other human interferons against HCV RNA replicons and related viruses. Antivir. Res..

[30] Garcia-Sastre A. (2017). Ten strategies of interferon evasion by viruses. Cell Host Microbe.

[31] Schoggins J.W., MacDuff D.A., Imanaka N., Gainey M.D., Shrestha B., Eitson J.L., Mar K.B., Richardson R.B., Ratushny A.V., Litvak V. (2014). Pan-viral specificity of IFN-induced genes reveals new roles for cGAS in innate immunity. Nature.

[32] Zhu C., Xiao F., Hong J., Wang K., Liu X., Cai D., Fusco D.N., Zhao L., Jeong S.W., Brisac C. (2015). EFTUD2 is a novel innate immune regulator restricting hepatitis C virus infection through the RIG-I/MDA5 Pathway. J. Virol..

[33] Zhou X., Michal J.J., Zhang L., Ding B., Lunney J.K., Liu B., Jiang Z. (2013). Interferon induced IFIT family genes in host anitiviral defense. Int. J. Biol. Sci..

[34] Seo J.Y., Yaneva R., Cresswell P. (2011). Viperin: A multifunctional, interferon-inducible protein that regulates virus replication. Cell Host Microbe.

[35] Gizzi A.S., Grove T.L., Arnold J.J., Jose J., Jangra R.K., Garforth S.J., Du Q., Cahill S.M., Dulyaninova N.G., Love J.D. (2018). A naturally occurring antiviral ribonucleotide encoded by the human genome. Nature.

[36] Dumbrepatil A.B., Ghosh S., Zegalia K.A., Malec P.A., Hoff J.D., Kennedy R.T., Marsh E.N.G. (2019). Viperin interacts with the kinase IRAK1 and the E3 ubiquitin ligase TRAF6, coupling innate immune signaling to antiviral ribonucleotide synthesis. J. Biol. Chem..

[37] Panayiotou C., Lindqvist R., Kurhade C., Vonderstein K., Pasto J., Edlund K., Upadhyay A.S., Overby A.K. (2018). Viperin restricts Zika virus and tick-borne encephalitis virus replication by targeting NS3 for proteasomal degradation. J. Virol..

[38] Vonderstein K., Nilsson E., Hubel P., Nygard Skalman L., Upadhyay A., Pasto J., Pichlmair A., Lundmark R., Overby A.K. (2017). Viperin targets flavivirus virulence by inducing assembly of noninfectious capsid particles. J. Virol..

[39] Szretter K.J., Brien J.D., Thackray L.B., Virgin H.W., Cresswell P., Diamond M.S. (2011). The interferon-inducible gene viperin restricts West Nile virus pathogenesis. J. Virol..

[40] Morales D.J., Lenschow D.J. (2013). The antiviral activities of ISG15. J. Mol. Biol..

[41] Bielefeldt-Ohmann H., Smirnova N.P., Tolnay A.E., Webb B.T., Antoniazzi A.Q., Van Campen H., Hansen T.R. (2012). Neuro-invasion by a ‘Trojan Horse’ strategy and vasculopathy during intrauterine flavivirus infection. Int. J. Exp. Pathol..

[42] Dai J., Pan W., Wang P. (2011). ISG15 facilitates cellular antiviral response to dengue and West Nile virus infection in vitro. Virol. J..

[43] Hewitt E.W., Lehner P.J. (2003). The ABC-transporter signature motif is required for peptide translocation but not peptide binding by TAP. Eur. J. Immunol..

[44] Liu S.Y., Sanchez D.J., Aliyari R., Lu S., Cheng G. (2012). Systematic identification of type I and type II interferon-induced antiviral factors. Proc. Natl. Acad. Sci. USA.

[45] Quicke K.M., Suthar M.S. (2013). The innate immune playbook for restricting West Nile virus infection. Viruses.

[46] Uddin M.J., Suen W.W., Prow N.A., Hall R.A., Bielefeldt-Ohmann H. (2015). West Nile virus challenge alters the transcription profiles of innate immune genes in rabbit peripheral blood mononuclear cells. Front. Vet. Sci..

[47] Uddin M.J., Suen W.W., Bosco-Lauth A., Hartwig A.E., Hall R.A., Bowen R.A., Bielefeldt-Ohmann H. (2016). Kinetics of the West Nile virus induced transcripts of selected cytokines and Toll-like receptors in equine peripheral blood mononuclear cells. Vet. Res..

[48] McGuckin Wuertz K., Treuting P.M., Hemann E.A., Esser-Nobis K., Snyder A.G., Graham J.B., Daniels B.P., Wilkins C., Snyder J.M., Voss K.M. (2019). STING is required for host defense against neuropathological West Nile virus infection. PLoS Pathog..

[49] Ding Q., Gaska J.M., Douam F., Wei L., Kim D., Balev M., Heller B., Ploss A. (2018). Species-specific disruption of STING-dependent antiviral cellular defenses by the Zika virus NS2B3 protease. Proc. Natl. Acad. Sci. USA.

[50] Robbins G.R., Truax A.D., Davis B.K., Zhang L., Brickey W.J., Ting J.P. (2012). Regulation of class I major histocompatibility complex (MHC) by nucleotide-binding domain, leucine-rich repeat-containing (NLR) proteins. J. Biol. Chem..

[51] Guo X., Liu T., Shi H., Wang J., Ji P., Wang H., Hou Y., Tan R.X., Li E. (2015). Respiratory syncytial virus infection upregulates NLRC5 and Major Histocompatibility Complex Class I expression through RIG-I induction in airway epithelial cells. J. Virol..

[52] Ranjan P., Singh N., Kumar A., Neerincx A., Kremmer E., Cao W., Davis W.G., Katz J.M., Gangappa S., Lin R. (2015). NLRC5 interacts with RIG-I to induce a robust antiviral response against influenza virus infection. Eur. J. Immunol..

[53] Wong E., Xu R.H., Rubio D., Lev A., Stotesbury C., Fang M., Sigal L.J. (2018). Migratory dendritic cells, group 1 innate lymphoid cells, and inflammatory monocytes collaborate to recruit NK cells to the virus-infected lymph node. Cell Rep..

[54] Muller M., Carter S., Hofer M.J., Campbell I.L. (2010). Review: The chemokine receptor CXCR3 and its ligands CXCL9, CXCL10 and CXCL11 in neuroimmunity—A tale of conflict and conundrum. Neuropathol. Appl. Neurobiol..

[55] Srivastava R., Khan A.A., Chilukuri S., Syed S.A., Tran T.T., Furness J., Bahraoui E., BenMohamed L. (2017). CXCL10/CXCR3-dependent mobilization of herpes simplex virus-specific CD8^+^ T_EM_ and CD8^+^ T_RM_ cells within infected tissues allows efficient protection against recurrent herpesvirus infection and disease. J. Virol..

[56] Fujino M., Sato H., Okamura T., Uda A., Takeda S., Ahmed N., Shichino S., Shiino T., Saito Y., Watanabe S. (2017). Simian immunodeficiency virus targeting of CXCR3^+^ CD4^+^ T Cells in secondary lymphoid organs is associated with robust CXCL10 expression in monocyte/macrophage subsets. J. Virol..

[57] Lu Y., Lin L.Y., Tan J.G., Deng H.P., Li X.H., Zhang Z., Li Y., Zhou Z., Xu X., Xie X. (2017). A correlation study between gene polymorphism of Th cell expressed chemokine receptor CXCR3 and its ligand levels with HCV infection prognosis. Eur. Rev. Med. Pharmacol. Sci..

[58] Oelmann E., Herbst H., Zuhlsdorf M., Albrecht O., Nolte A., Schmitmann C., Manzke O., Diehl V., Stein H., Berdel W.E. (2002). Tissue inhibitor of metalloproteinases 1 is an autocrine and paracrine survival factor, with additional immune-regulatory functions, expressed by Hodgkin/Reed-Sternberg cells. Blood.

[59] Zanotti L., Angioni R., Cali B., Soldani C., Ploia C., Moalli F., Gargesha M., D’Amico G., Elliman S., Tedeschi G. (2016). Mouse mesenchymal stem cells inhibit high endothelial cell activation and lymphocyte homing to lymph nodes by releasing TIMP-1. Leukemia.

[60] Jones R.S., Tu C., Zhang M., Qu J., Morris M.E. (2019). Characterization and proteomic-transcriptomic investigation of monocarboxylate transporter 6 knockout mice: evidence of a potential role in glucose and lipid metabolism. Mol. Pharmacol..

[61] Oudshoorn D., Van Boheemen S., Sanchez-Aparicio M.T., Rajsbaum R., Garcia-Sastre A., Versteeg G.A. (2012). HERC6 is the main E3 ligase for global ISG15 conjugation in mouse cells. PLoS ONE.

[62] Afgan E., Baker D., Batut B., Van den Beek M., Bouvier D., Cech M., Chilton J., Clements D., Coraor N., Gruning B.A. (2018). The Galaxy platform for accessible, reproducible and collaborative biomedical analyses: 2018 update. Nucleic Acids Res..

[63] Hall R.A., Broom A.K., Hartnett A.C., Howard M.J., Mackenzie J.S. (1995). Immunodominant epitopes on the NS1 protein of MVE and KUN viruses serve as targets for a blocking ELISA to detect virus-specific antibodies in sentinel animal serum. J. Virol. Methods.

[64] Prow N.A., Tan C.S., Wang W., Hobson-Peters J., Kidd L., Barton A., Wright J., Hall R.A., Bielefeldt-Ohmann H. (2013). Natural exposure of horses to mosquito-borne flaviviruses in south-east Queensland, Australia. Int. J. Environ. Res. Public Health.

