# Supplementary material for: An Acute Stress Model in New Zealand White Rabbits Exhibits Altered Immune Response to Infection with West Nile Virus
Source: Pathogens. 2019 Oct 18;8(4):195. doi: 10.3390/pathogens8040195 (PMC6963736; doi:10.3390/pathogens8040195)
Supplement: Supplementary file 1 [file pathogens-08-00195-s001.pdf]

Supplementary material

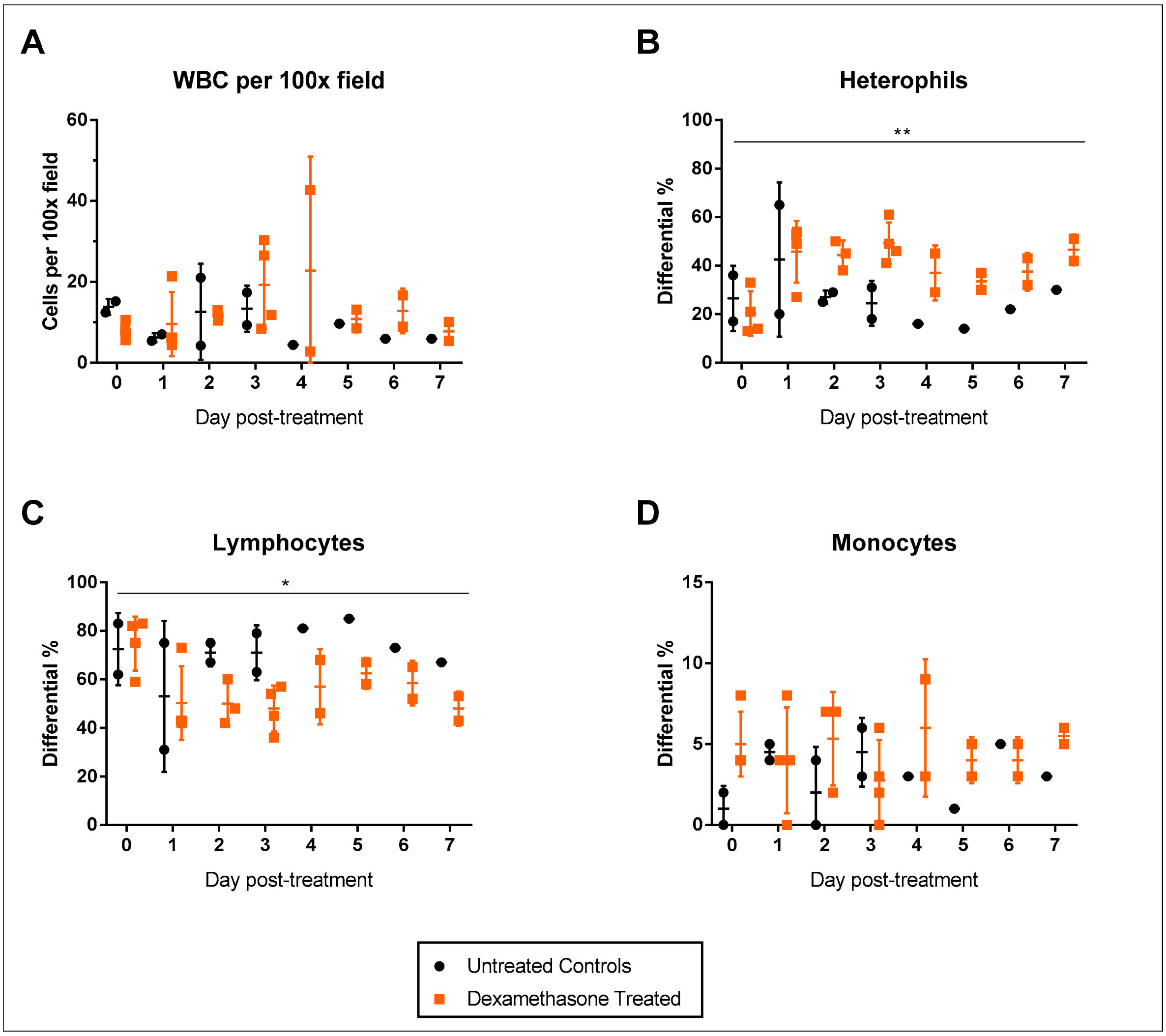

Figure S1. Effect of bolus administration of dexamethasone on hematologic profile in uninfected rabbits. WBC, white blood cell; *, p = 0.01-0.05; **, p = 0.001 - 0.01; ***, p = 0.0001 - 0.001, ****, p < 0.0001.

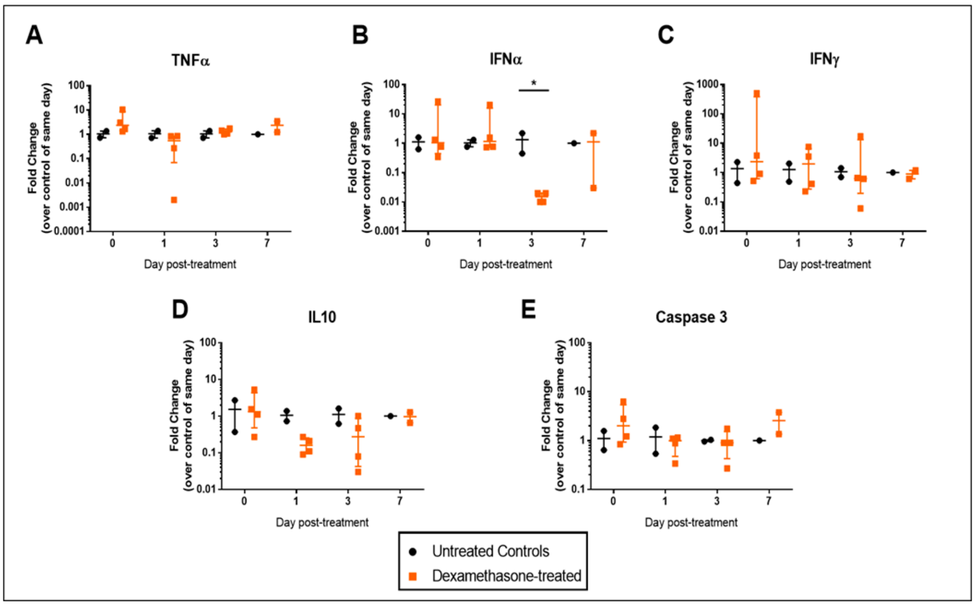

Figure S2. Effect of dexamethasone treatment on mRNA expression for a select set of cytokines in whole blood, as determined by qRT-PCR. A statistically significant effect of the treatment is only seen on the IFN-α transcript on day 3 pdt, with expression restored by day 7 pdt. *, p = 0.01-0.05; **, p = 0.001 - 0.01; ***, p = 0.0001 - 0.001, ****, p < 0.0001.

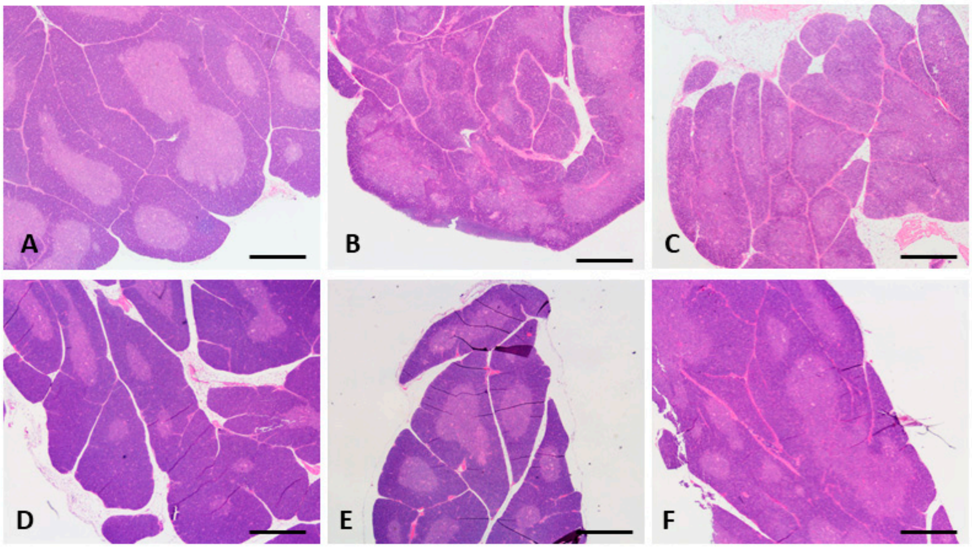

Figure S3. Effect of bolus administration of dexamethasone on thymic morphology in young rabbits. (**A**)Thymus from mock-treated animal terminate on day 3 pdt, (**B & C**) Thymus from the two dexamethasone-treated animals terminated on day 3 pdt, (**D**) Thymus from mock-treated animal terminated on day 7 post injection, (**E & F**) Thymus from the two dexamethasone-treated animals terminated on day 7 dpt. H&E stain. Scale bar = 200 μm.

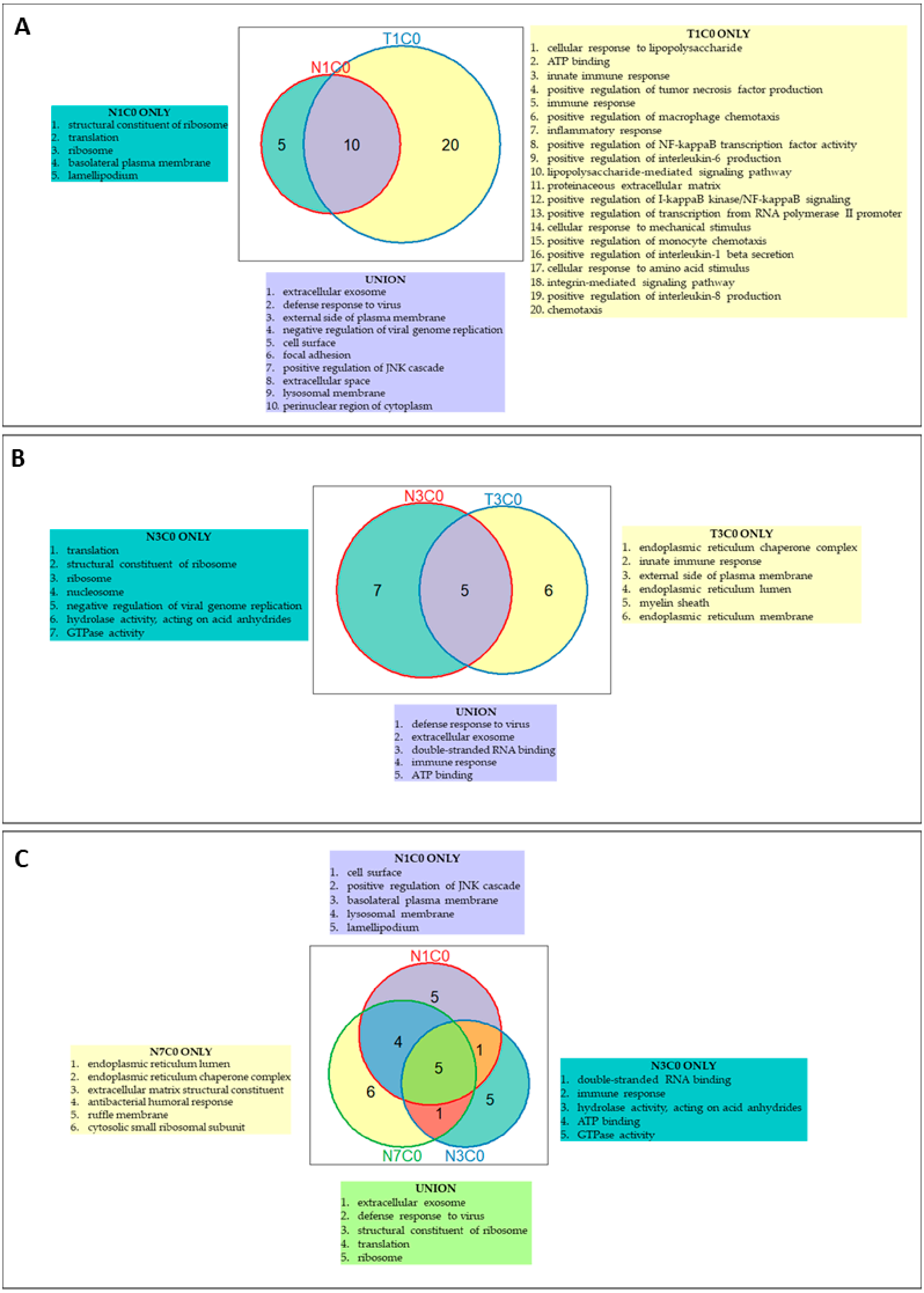

Figure S4. Enriched GO terms (adjusted p-value < 0.05) from each of the pairwise comparisons were analyzed for commalities and differences using Venn diagrams (Vennerable, R package).

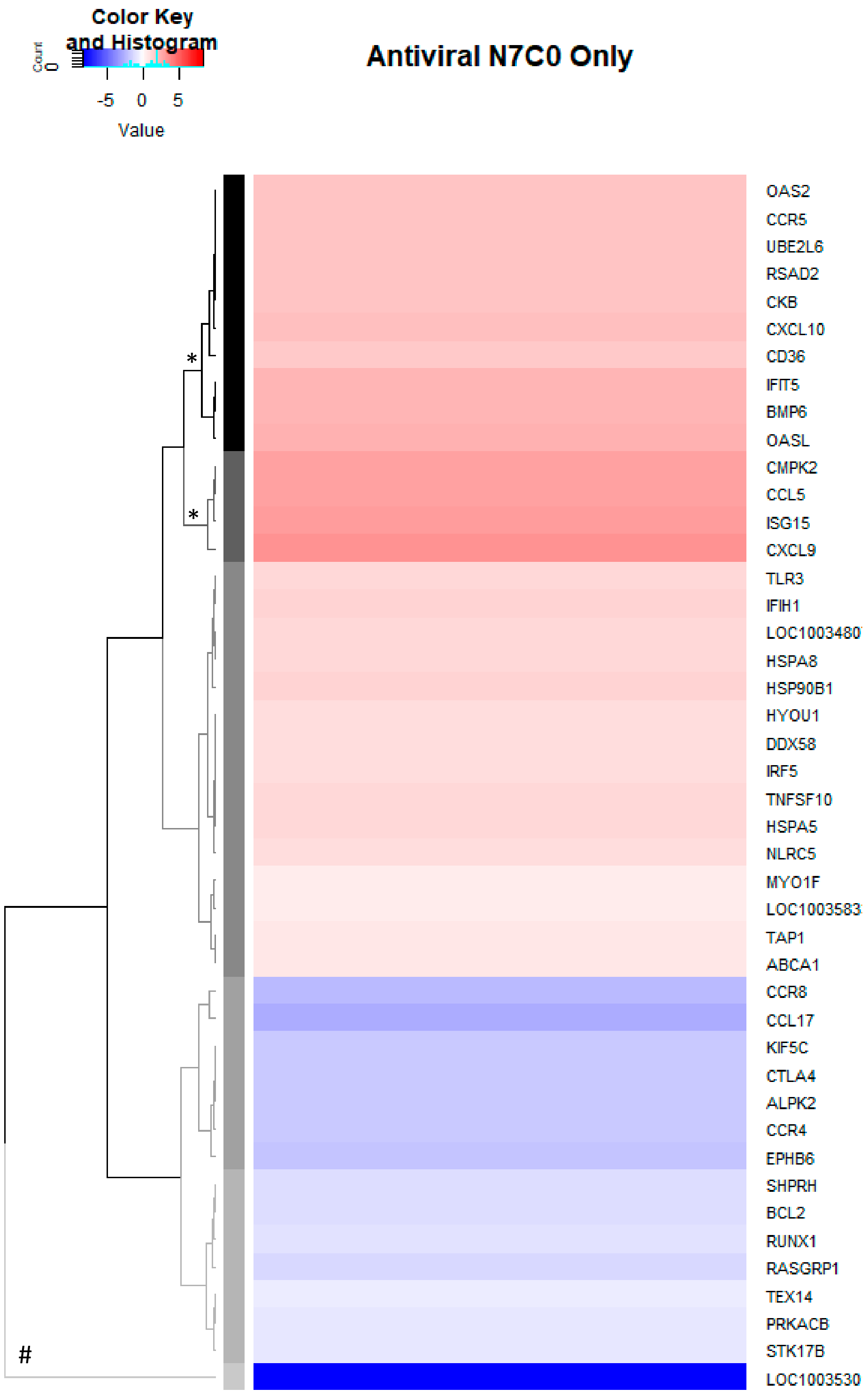

Figure S5. DEGs associated with GO terms relevant to antiviral immune responses on day 7 p.i.. The symbols * and # represent the top up- and down-regulated clusters of genes, respectively. The grey scale dendrogram branches and the corresponding sidebar in each heatmap represent the different clusters. Clusters with symbols * and # indicate top up- and down-regulated clusters, respectively. The blue-red scalebar in the top left corner of each heatmap corresponds to the color scale of the heatmap, indicating the logFC for each gene. The light blue histogram within these scalebars indicate the frequency of genes at each level of logFC.

Table S1. Top clusters of antiviral DEGs on day 7 p.i. (summary of Figure S5)

		**Antiviral DEGs expressed in mock-treated rabbits**
**Day 7 p.i.**	**Up**	CXCL9#<br>ISG15<br>CCL15<br>CMPK2
		OAS2<br>CCR5<br>UBE2L6<br>RSAD2<br>CKB<br>CXCL10<br>CD36<br>IFIT5<br>BMP6<br>OASL
	**Down**	LOC100353012<br>(ATP-binding cassette subfamily A member 3)
		CCR8<br>CCL17<br>KIF5C<br>CTLA4<br>ALPK2<br>CCR4<br>EPHB6

Note: only top 2 clusters of upregulated and downregulated DEGs are summarized in this table (hierarchical clustering was performed based on the log-fold change of the genes). Clusters are demarcated by dashed borders.
